# Musculoskeletal manifestations associated with transthyretin-mediated (ATTR) amyloidosis: a systematic review

**DOI:** 10.1186/s12891-023-06853-5

**Published:** 2023-09-22

**Authors:** Emre Aldinc, Courtney Campbell, Finn Gustafsson, Abigail Beveridge, Richard Macey, Laura Marr, Catherine Summers, Dafang Zhang

**Affiliations:** 1 Alnylam Pharmaceuticals, Cambridge, MA US; 2 Baylor Scott & White Heart and Vascular Hospital, Dallas, TX US; 3 Rigshopsitalet, University of Copenhagen, Copenhagen, Denmark; 4 Adelphi Values PROVE™, Bollington, UK; 5 Brigham and Women’s Hospital, Boston, MA US

## Abstract

**Background:**

Hereditary and wild-type transthyretin-mediated (ATTRv and ATTRwt) amyloidoses result from the misfolding of transthyretin and aggregation of amyloid plaques in multiple organ systems. Diagnosis of ATTR amyloidosis is often delayed due to its heterogenous and non-specific presentation. This review investigates the association of musculoskeletal (MSK) manifestations with ATTR amyloidosis and the delay from the onset of these manifestations to the diagnosis of ATTR amyloidosis.

**Methods:**

This systematic review utilized Medline and EMBASE databases. Search criteria were outlined using a pre-specified patient, intervention, comparator, outcome, time, study (PICOTS) criteria and included: amyloidosis, ATTR, and MSK manifestations. Publication quality was assessed utilizing Joanna Briggs Institute (JBI) critical appraisal checklists.

The search initially identified 7,139 publications, 164 of which were included. PICOTS criteria led to the inclusion of epidemiology, clinical burden and practice, pathophysiology, and temporality of MSK manifestations associated with ATTR amyloidosis. 163 publications reported on ATTR amyloidosis and MSK manifestations, and 13 publications reported on the delay in ATTR amyloidosis diagnosis following the onset of MSK manifestations.

**Results:**

The MSK manifestation most frequently associated with ATTR amyloidosis was carpal tunnel syndrome (CTS); spinal stenosis (SS) and osteoarthritis (OA), among others, were also identified. The exact prevalence of different MSK manifestations in patients with ATTR amyloidosis remains unclear, as a broad range of prevalence estimates were reported. Moreover, the reported prevalence of MSK manifestations showed no clear trend or distinction in association between ATTRv and ATTRwt amyloidosis.

MSK manifestations precede the diagnosis of ATTR amyloidosis by years, and there was substantial variation in the reported delay to ATTR amyloidosis diagnosis. Reports do suggest a longer diagnostic delay in patients with ATTRv amyloidosis, with 2 to 12 years delay in ATTRv versus 1.3 to 1.9 years delay in ATTRwt amyloidosis.

**Conclusion:**

These findings suggest that orthopedic surgeons may play a role in the early diagnosis of and treatment referrals for ATTR amyloidosis. Detection of MSK manifestations may enable earlier diagnosis and administration of effective treatments before disease progression occurs.

**Supplementary Information:**

The online version contains supplementary material available at 10.1186/s12891-023-06853-5.

## Introduction

### Background

Systemic amyloidoses are protein-misfolding diseases characterized by the aggregation and deposition of amyloid plaques in multiple organ systems [1, 2]. Transthyretin-mediated (ATTR) amyloidosis is caused by misfolding of the precursor protein transthyretin (TTR) [1, 2]. There are two types of ATTR amyloidoses, variant (ATTRv) [also known as hereditary or hATTR] and wild-type (ATTRwt) [2, 3]. In ATTRv amyloidosis, point variants in the *TTR* gene lead to destabilization and dissociation of TTR from its native tetrameric conformation, and subsequent aggregation as amyloid fibrils [4]. In ATTRwt amyloidosis, wild-type, non-variant TTR dissociates, and amyloid aggregation occurs [4]. ATTRwt and ATTRv amyloidoses overlap in their clinical presentation, and therefore, definitive distinction relies on *TTR* gene sequencing in suspected patients [2]. ATTRv amyloidosis affects approximately 50,000 people worldwide. While the exact prevalence of ATTRwt amyloidosis is unknown, it is thought to be more prevalent than ATTRv amyloidosis [1, 5].

ATTR amyloidosis is a heterogeneous, multisystem disease in which a significant proportion of patients develop a mixed phenotype of polyneuropathy (PN) and cardiomyopathy (CM) [2, 5, 6]. The disease is rapidly progressive; ATTRv and ATTRwt amyloidoses have a median survival of 4.7 years and 3.6 years after diagnosis, respectively, and disease progression substantially negatively impacts quality of life [5, 7, 8]. Diagnosis can be difficult or delayed due to the heterogenous, non-specific nature of ATTR amyloidosis and symptom overlap with other diseases [9–11]. Various musculoskeletal (MSK) manifestations, such as carpal tunnel syndrome (CTS), spinal stenosis (SS), osteoarthritis (OA), and others, have been reported in patients with ATTR amyloidosis [1]. Importantly, these MSK manifestations have been shown to precede the diagnosis of the disease by years [1, 4, 11].

### Rationale

The typical patient journey before being diagnosed with ATTR amyloidosis is lengthy and involves consulting numerous physicians from different specialties [2, 11]. Consequently, ATTR amyloidosis may remain undetected, and treatment is often delayed until the disease progresses to an advanced stage. This diagnostic delay increases patient disability and morbidity, whereas earlier therapeutic intervention can attenuate disease progression and worsening in patient quality of life. [2]. Enabling earlier diagnosis of ATTR amyloidosis is critical to improving overall patient prognosis [1]. Various MSK manifestations have been reported in the literature to be associated with ATTR amyloidosis. Additionally, certain manifestations, such as CTS, symptoms of which can also be caused by the PN of ATTR amyloidosis, are already included among the early signs, which are considered ‘red flags’ for the disease.

This systematic review was conducted to investigate the association between ATTR amyloidosis and MSK manifestations, and to investigate the temporal association between the onset of MSK manifestations and ATTR amyloidosis diagnosis.

## Methods

### Search strategy and criteria

The protocol for this systematic review is registered on the international prospective register of systematic reviews (PROSPERO) from the National Institute for Health Research Database (www.crd.york.ac.uk/prospero; protocol no. CRD42022310956), and the PRISMA statement was adhered to [12].

An electronic database search was run on November 3, 2021 across two databases in Ovid®: Medline and EMBASE. No restriction on publication year was applied. Search strategies are detailed in Supplement 1.

Gray literature searches included hand searches of previously published systematic reviews and a review of conference proceedings from 2019 to 2021. Independent hand searches of conference proceedings were conducted for the American Association for Hand Surgery (AAHS), American Society for Surgery of the Hand (ASSH), European Society of Cardiology (ESC), European ATTR amyloidosis meeting (EU-ATTR), Federation of European Societies for the Surgery of the Hand (FESSH), International Federation of Societies for Surgery of the Hand (IFSSH), International Society of Amyloidosis (ISA), and the International Society for Pharmacoeconomics and Outcomes Research (ISPOR). Conferences of interest that were not independently hand searched, given that the EMBASE electronic database search already captured their proceedings, included the American College of Cardiology (ACC), Heart Failure Society of America (HFSA), and the Peripheral Nerve Society (PNS).

### Inclusion and exclusion criteria

The inclusion and exclusion criteria were pre-defined in a patient, intervention, comparator, outcome, time, study (PICOTS) table during protocol development (Supplement 2). These included outcomes related to the epidemiology, pathophysiology, temporal association (the time from the diagnosis of the MSK manifestation(s) to the diagnosis of ATTR amyloidosis), clinical burden, and current clinical practice related to MSK manifestations associated with ATTR amyloidosis. Publications reporting data only from patients diagnosed with amyloidoses other than ATTR amyloidosis were excluded, as were publications reporting on outcomes related to MSK manifestations outside of an ATTR amyloidosis context and/or publications reporting separately on either ATTR amyloidosis or MSK manifestations. Case series were included, while case reports involving individual patients were excluded [13]; for the list of those case reports by MSK manifestation, refer to Supplement 3.

All abstracts and full texts included were screened by two separate reviewers. Conflicts on inclusion or exclusion were resolved by a third senior reviewer.

**Fig. 1:**
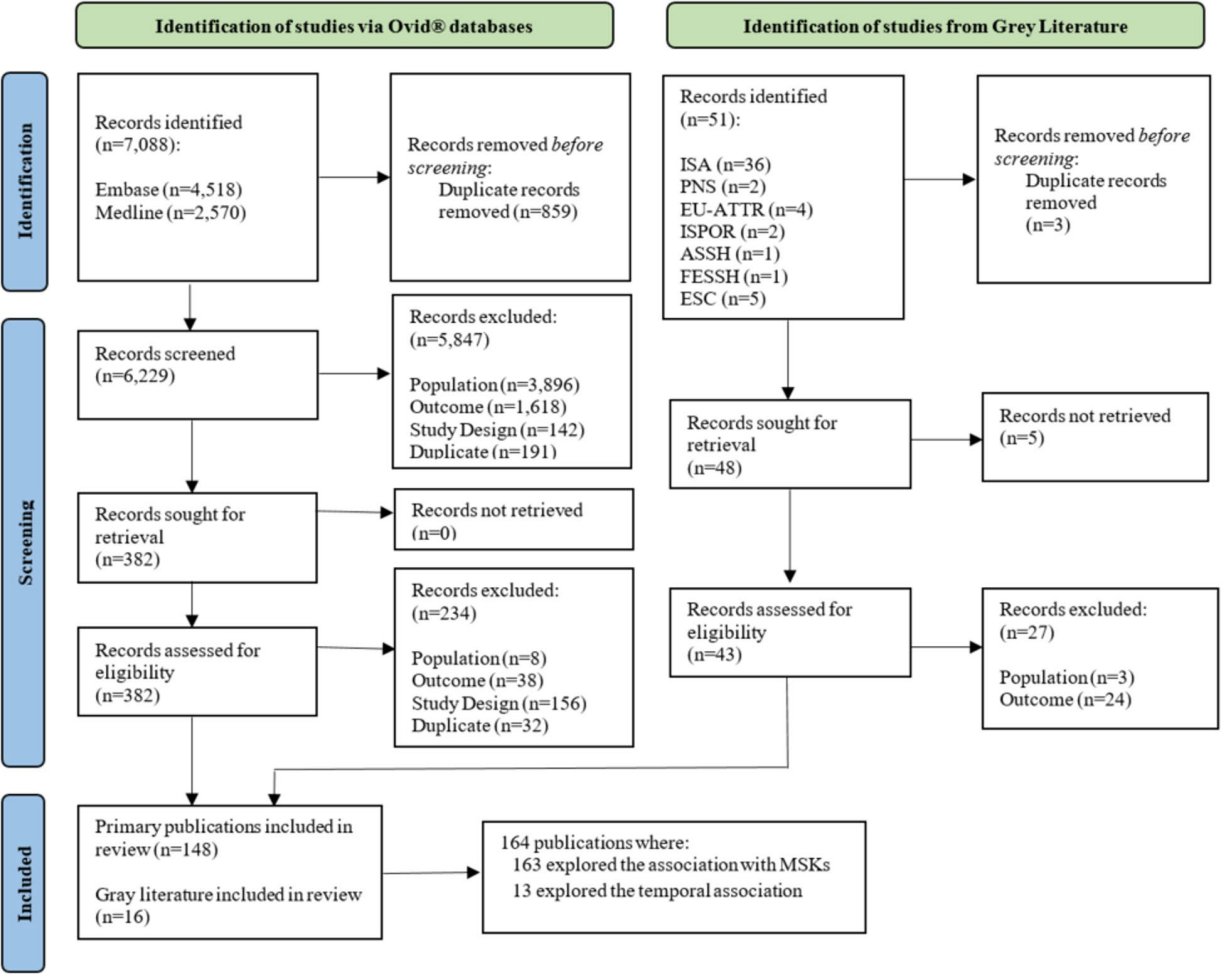
PRISMA flow diagram of the study identification and screening process. ASSH = American Society for Surgery of the Hand; ESC = European Society of Cardiology; EU-ATTR = European transthyretin-mediated amyloidosis meeting; FESSH = Federation of European Societies for Surgery of the Hand; n = number; ISA = International Society of Amyloidosis; ISPO = International Society for Pharmacoeconomics and Outcomes Research; PNS = Peripheral Nerve Society; SLR = systematic literature review

Of the 7,139 publications identified, 164 publications were included in the analysis, as shown in the PRISMA diagram, (Fig. 1). Importantly, authors of the publications included approached the association between MSK manifestations and presence of ATTR amyloidosis differently. For example, some authors investigated MSK manifestations in patients with a confirmed diagnosis of ATTR amyloidosis, whereas other authors investigated the presence of ATTR amyloidosis in patients who had undergone treatment for MSK manifestations or who were diagnosed with a MSK manifestation, presented in Table 1.

**Table 1:** Cross-tabulation of the number of publications investigating the association between ATTR amyloidosis and MSK manifestations, and the direction of the association reported

MSK manifestation reported in the included publications	Total number of publications identified	Number of publications that reported ATTR amyloidosis in patients with MSK manifestations	Number of publications that reported MSK manifestations in patients with ATTR amyloidosis
*Publications where one MSK manifestation was reported*			
Carpal tunnel syndrome	109	22	87
Spinal stenosis	9	2	7
Osteoarthritis	8	8	0
Trigger finger	1	1	0
*Publications where more than one MSK manifestation was reported*			
Carpal tunnel syndrome and spinal stenosis	20	3	17
Carpal tunnel syndrome, spinal stenosis, and trigger finger	2	0	2
Carpal tunnel syndrome, spinal stenosis, and osteoarthritis	2	0	2
Carpal tunnel syndrome, spinal stenosis, and biceps tendon rupture	2	2	0
Hip arthroplasty and knee arthroplasty (osteoarthritis)	2	0	2
Other^a^	8	2	6

^a^ATTR amyloidosis in patients where more than one musculoskeletal manifestation was reported and multiple musculoskeletal manifestations in patients with ATTR amyloidosis, refer to Table 7

**Fig. 2:**
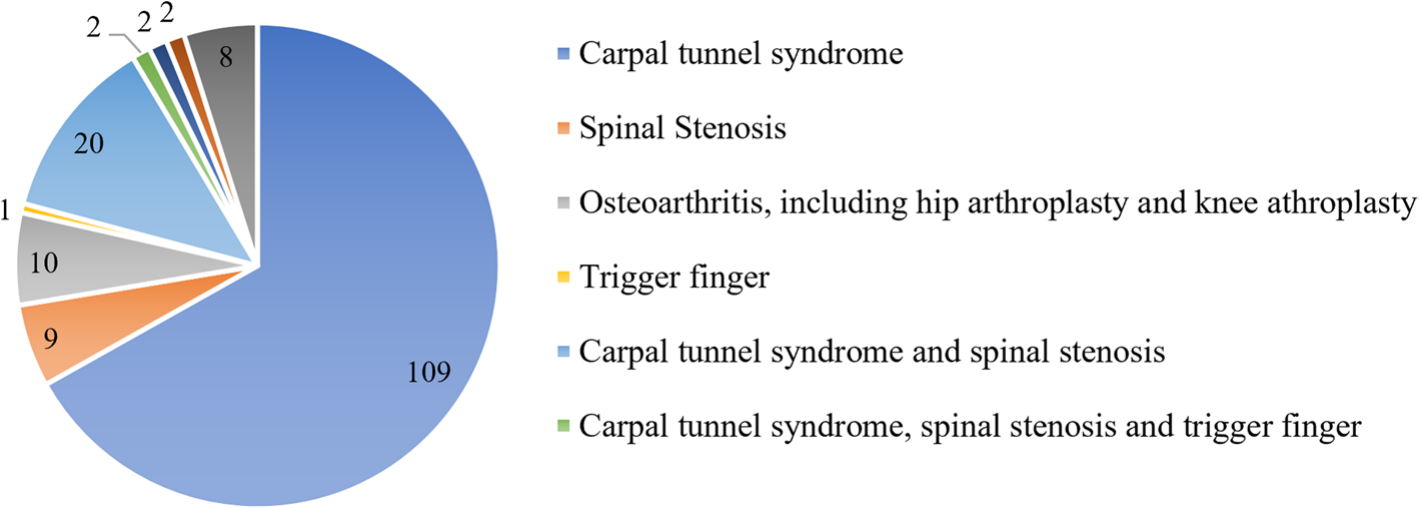
Number of publications reporting on various MSK manifestations associated with ATTR amyloidosis. Eight other publications reported on the association between ATTR amyloidosis and several different MSK manifestations in various combinations, the details of which are reported in Table 7

**Fig. 3:**
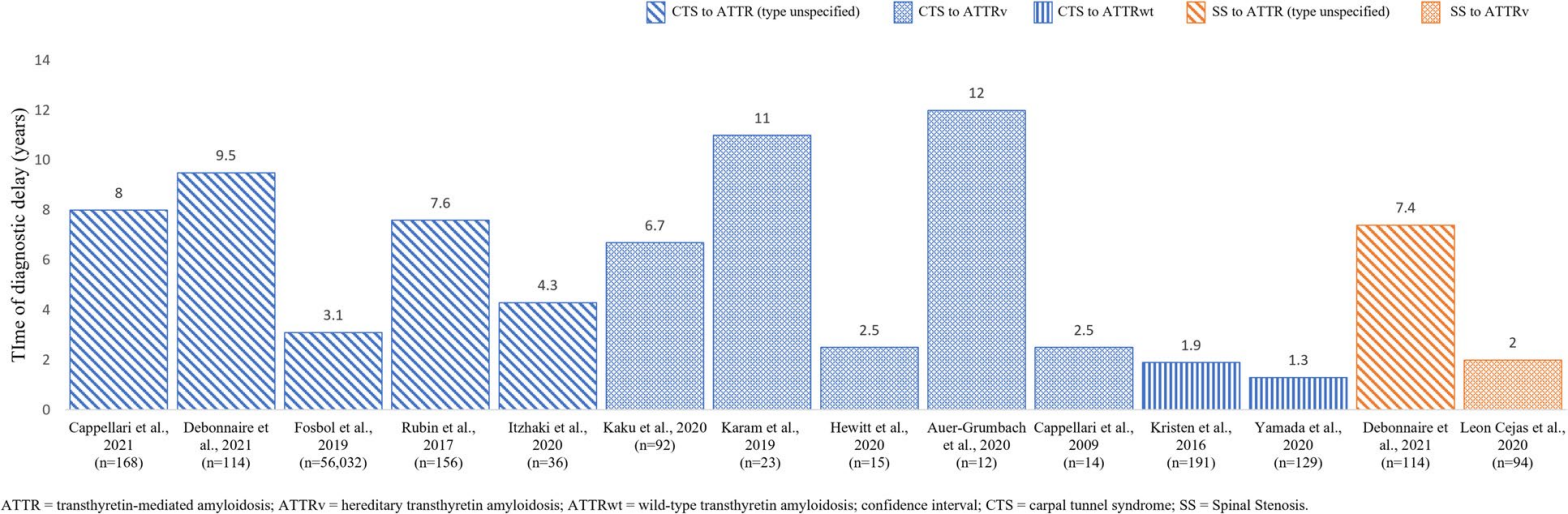
Time between MSK symptom onset and ATTR amyloidosis diagnosis

One hundred sixty-three publications examined the association between MSK manifestations and ATTR amyloidosis (Fig. 2 provides an overview of studies and Tables 2, 3, 4, 5, 6 and 7 provide study details), and 13 publications investigated the temporal association between MSK manifestations and ATTR amyloidosis (Fig. 3 with study details reported in Table 8). One publication reported only on the temporal delay and did not report on the association between MSK manifestations and ATTR amyloidosis.

**Table 2:** Carpal tunnel syndrome in patients with ATTR amyloidosis

Publication	Study design	Research population	Prevalence	Additional statistics	Information relating to and/or confirming diagnosis^a^
*Carpal tunnel syndrome in ATTR amyloidosis*					
Abe *et al.*, 2021 [14]	Case Series	90 patients with ATTR amyloidosis (type unspecified)	7.8% with CTS	NR	MSK biopsy and staining (tenosynovial tissue within the transverse carpal ligament) Other biopsy and staining MS Autopsy
		44 patients with ATTRv amyloidosis	18.2% with CTS	NR	
		106 patients with ATTRwt amyloidosis	34.9% with CTS	NR	
Akinboboye *et al.*, 2015 [15]	Cross-sectional survey	14 patients with ATTRv amyloidosis (Val122Ile point mutation)	14% with CTS	NR	Gene testing and/or point mutation testing in patients Point mutations: Val122Ile
Ando *et al.*, 2021 [16]	Case series	1,937 patients with ATTR amyloidosis (type unspecified)	ATTRwt: 17% with CTS	NR	MSK biopsy and staining Other biopsy and staining Myocardium, submucosa, skin (with fat) Gene and/or point mutation testing Point mutations: (V30M, p.TTR, V50M, P24S, A25T, V28M, V28S, V30A, F33V, A36D, A45D, G47V, G47R, T49I, T49S, S50I, S50R, G53E, E54L, L55P, T59R, T60A, E61K, S77Y, K80R, G83R, E89K, E89Q, V94G, A97G, R104H, I107V, Y114C, Y114S, and V122I) MS
			ATTRv: 6.8% with CTS	NR	
Arevalo *et al.*, 2020 [17]	Database/registry	12,745 patients with cardiac ATTR amyloidosis (type unspecified)	60 (0.5%) with CTS	Multivariate linear regression adjustment for confounders of age, gender, race, hypothyroidism, and diabetes mellitus showed a significant relationship between cardiac ATTR amyloidosis and CTS OR=4.31, 2.46–7.56 (OR 95% CI), p<0.001	NR
Bishop *et al.*, 2018 [18]	Case series	52 patients with ATTR amyloidosis (type unspecified)	27 (51.9%) with CTS	NR	Cohort 1: Biopsy and staining, MS
		67 patients with ATTRv amyloidosis	NR	4.57 (RR) with 2.58–8.09 (95% CI) of CTS leading to ATTR diagnostic delay	Cohort 2: Biopsy and staining
Bukhari *et al.*, 2020 [19]	Case series	45 patients with ATTR amyloidosis (type unspecified)	62% with CTS, p<0.01	NR	LV wall thickening ^99m^PYP/DPD imaging
Bukhari *et al.*, 2021 [20]	Case series	125 Caucasian patients with ATTR amyloidosis (94% ATTRwt; 6% ATTRv)	76% with bilateral CTS	NR	^99m^PYP/DPD imaging Gene and/or point mutation testing Point mutation s: pV50M, pT80A
		20 African American patients with ATTR amyloidosis (25% ATTRv; 75% ATTRwt)	81% with bilateral CTS	NR	
Cappellari *et al.*, 2009 [21]	Case series	14 patients with ATTRv amyloidosis	1 with CTS	NR	Biopsy and staining Gene and/or point mutation testing Point mutations: Val30Met, Phe64Leu, Asn124Ser, Glu89Gln, Val122Ile, Ile107Phe, Thr49Ala, Ser50Arg
ss Cappellari *et al.*, 2011 [22]	Case series	17 patients with ATTRv amyloidosis	3 (17.6%) with CTS	NR	Biopsy and staining Gene and/or point mutation testing Point mutation s: p.Val30Met, p.Arg34Thr, p.Thr49Ala, p.Ser50Arg, p.Phe64Leu, p.Glu89Gln, and p.Ile107Phe
Cappelli *et al.*, 2021 [23]	Cross-sectional^a^	168 patients with ATTR amyloidosis (type unspecified)	122 (73%) with CTS	NR	Point mutation testing: Point mutations: le68leu, Val122ile, other (unspecified)
Cerudelli *et al.*, 2019 [24]	Case series	81 patients with ATTR amyloidosis (type unspecified)	20% with CTS	NR	^99m^PYP/DPD imaging
Chen *et al.*, 2021 [25]	Case series	29 patients with ATTRv amyloidosis	17 (57.5%) with CTS	Prevalence by age: 15 (62.5%) aged 61(51–70) years old; 2 (40%) aged 42(30–48%) years old	Biopsy and staining LV wall thickening Point mutation testing
Choi *et al.*, 2020 [26]	Case series	135 patients with ATTR amyloidosis (type unspecified)	15 with CTS	NR	NR
Cipriani *et al.*, 2019 [27]	Case series	18 patients with ATTRwt amyloidosis	12 (66%) with CTS	NR	Biopsy and staining Gene and/or point mutation testing
Cortese *et al.*, 2014 [28]	Case series	150 patients with ATTRv amyloidosis	41% with CTS	NR	Gene and/or point mutation testing Point mutations: Val30Met, Glu89Gln, Phe64Leu, Ile68Leu
Minutoli *et al.*, 2010 [29]	Case series	16 patients with ATTRv amyloidosis	3 (18.8%) with CTS	NR	LV wall thickening Gene and/or point mutation testing Point mutations: Glu89Gln, Phe64Leu, Thr49A, Gly6Ser ^99m^PYP/DPD imaging
Du *et al.*, 2021 [30]	Case series	54 patients with ATTRv amyloidosis	14 (25.9%) with CTS	NR	Biopsy and staining Gene and/or point mutation testing Point mutations: Val30Met, Ala97Ser
		12 patients with ATTRv amyloidosis (Val30Met point mutation)	2 (16.7%) with CTS	NR	
		7 patients with ATTRv amyloidosis (Ala97Ser point mutation)	4 (57.1%) with CTS	NR	
Durmus *et al.*, 2014 [31]	Case series	14 patients with ATTRv amyloidosis	5 with CTS	NR	Gene and/or point mutation testing Point mutations: Val30Met, Glu89Gln, Gly53Glu, Glu74Gly, Gly47Glu, Glu109Gly
Durmus *et al.*, 2012 [32]	Case series	10 patients with ATTRv amyloidosis	1 with CTS	NR	Gene and/or point mutation testing Point mutations: Glu89Gln
Erdogan *et al.*, 2020 [33]	Database/registry	44 patients with ATTRv amyloidosis	10 with CTS	NR	Biopsy and staining Gene and/or point mutation testing Point mutations: Val30Met, Glu89Gln, Gly47Ala, Gly47Glu, Gly53Glu, Glu54Gly, Val32Ala, Asp18Asn and Ala45Thr.
Eriksson *et al.*, 2009 [34]	Case series	33 patients with ATTR amyloidosis (ATTRv and ATTRwt)	3 with CTS (all ATTRwt)	NR	MSK biopsy and staining with Congo red (unspecified tissues obtained at CTRS) Gene and/or point mutation testing
Gabrovesk *et al.*, 2019 [35]	Case series	96 patients with ATTR amyloidosis (76% ATTRv [Val122Ile]; 22% ATTRwt; 1% ATTRv [Asp18Asn]; 1% ATTRv [Glu54Gln])	46% with CTS	NR	Biopsy and staining Gene and/or point mutation testing Point mutations: Val122Ile
Gagliardi *et al.*, 2018 [36]	Case series	82 patients with ATTRwt amyloidosis	30 (37%) with CTS	NR	Biopsy and staining LV wall thickening Gene and/or point mutation testing Point mutations: Ile68Leu
		67 patients with ATTRv amyloidosis	29 (43%) with CTS	NR	
Galat *et al.*, 2016 [37]	Case series	17 patients with ATTR amyloidosis (13 ATTRwt; 1 ATTRv; 3 unspecified)	5 with CTS	NR	Biopsy and staining Gene and/or point mutation testing Point mutations: Val122I ^99m^PYP/DPD imaging
Gawor *et al.*, 2018 [38]	Case series	4 patients with ATTRv amyloidosis (3 Phe33Leu; 1 Ala81Val)	2 with CTS	NR	LV wall thickening Gene and/or point mutation testing Point mutations: Phe33Leu
Gawor *et al.*, 2019 [39]	Case series	6 patients with ATTRwt amyloidosis	4 (75%) with CTS	NR	LV wall thickening Gene and/or point mutation testing ^99m^PYP/DPD imaging
Gawor *et al.*, 2020 [40]	Case series	58 patients with ATTRv amyloidosis	4 with CTS	NR	NR
Gentile *et al.*, 2020 [41]	Case series	9 patients with ATTRv amyloidosis	6 (67%) with CTS	NR	Gene and/or point mutation testing Point mutations: V122I and E89Q ^99m^PYP/DPD imaging
Goena *et al.*, 2021 [42]	Case series	181 patients with suspected ATTR amyloidosis (type unspecified)	NR	CTS as a predictor of ATTR diagnosis: OR=15.02; 95% CI (3.66–61.65); p<0.001	^99m^PYP/DPD imaging
Gospodinova *et al.*, 2015 [43]	Cross-sectional	40 patients with ATTRv amyloidosis (Glu89Gln point mutation)	17 (42.5%) with CTS	NR	NR
Hewitt *et al.*, 2020 [44]	Case series	15 patients with ATTRv amyloidosis (T60A point mutation)	5 (33%) with CTS	NR	Biopsy and staining Point mutation testing Point mutations: T60a
Hussain *et al.*, 2019 [45]	Case series	12 patients with ATTRv amyloidosis	1 with CTS	NR	Biopsy and staining Point mutation testing ^99m^PYP/DPD imaging
Jercan *et al.*, 2020 [46]	Case series	23 patients with ATTRv amyloidosis (18 with Glu54Gln point mutation)	7 (53%) with CTS	NR	Point mutation testing Point mutations: Glu54Gln
Kaku *et al.*, 2020 [47]	Case series	92 patients with ATTRv amyloidosis	72% with CTS	NR	NR
Kalinoski-Dubose *et al.*, 2020 [48]	Case series	26 patients with ATTRv amyloidosis (male)	84% with CTS	NR	LV wall thickening Point mutation testing Point mutations: V122I
		12 patients with ATTRv amyloidosis (female)	36% with CTS	NR	
Karam *et al.*, 2019 [49]	Case series	23 patients with ATTRv amyloidosis	17 with CTS	Age of CTS symptom onset 48.5(36–63) years old	Point mutation testing Point mutations: Val30Met, Glu89Gln, Gly53Glu, Glu54Gly, Gly47Glu
Keller *et al.*, 2021 (hATTR Compass study) [50]	Database/registry	28 patients with ATTRv amyloidosis (“rare unspecified” point mutations	30% with CTS	NR	Point mutation testing Point mutations: p.V142I, p.T80A, unspecified
		466 patients with ATTRv amyloidosis (V142I point mutation)	23% with CTS	NR	
		15 patients with ATTRv amyloidosis (T80A point mutation)	44% with CTS	NR	
Kessler *et al.*, 2019 [51]	Cross-sectional survey	68 patients with ATTR amyloidosis (type unspecified)	5 (5%) with CTS	17 patients received their diagnosis within 6 months of initial symptoms, average of 3.6 years until diagnosis reached	MSK biopsy and staining Other biopsy and staining Gene and/or point mutation testing ^99m^PYP/DPD imaging
Khella *et al.*, 2021 [52]	Database/registry	345 patients with ATTRv amyloidosis	90 (26%) with CTS	NR	Point mutation testing
Khella *et al.*, 2021 [53]	Database/registry	14 patients with ATTRv amyloidosis (p.V142l)	22% with CTS	NR	Point mutation testing Point mutations: p.V142I
Khella *et al.*, 2021 [54]	Database/registry	37 patients with ATTRv amyloidosis (p.V50M)	15% with CTS	NR	Point mutation testing
		42 patients with ATTRv amyloidosis (p.T80A)	35% with CTS	NR	Point mutations: p.V50M, p.T80A
Khella *et al.*, 2021 (hATTR Compass) [55]	Database/registry	321 patients with ATTRv amyloidosis (V142l/V122 point mutations)	20% with CTS	NR	NR
Kristen *et al.*, 2010 [56]	Case series	24 patients with ATTRwt amyloidosis	2 (8.3%) with CTS	NR	Gene testing
Kristen *et al.*, 2016 [57]	Case series	191 patients with ATTRwt amyloidosis	87 (48.3%) with CTS	NR	Gene testing
La Malfa *et al.*, 2019 [58]	Case series^b^	21 patients with ATTRwt amyloidosis	9.0% with CTS	NR	^99m^PYP/DPD imaging
Longhi *et al.*, 2014 [59]	Case series	260 patients with ATTR amyloidosis (type unspecified)	90 (35.0%) with CTS (18 ATTRwt; 72 ATTR unspecified)	NR	NR
Longhi *et al.*, 2015 [60]	Case series	5 patients with ATTRwt amyloidosis	2 with CTS	NR	LV wall thickening Gene testing ^99m^PYP/DPD imaging
Luigetii *et al.*, 2012 [61]	Case series	15 patients with ATTRv amyloidosis	13 (86%) with CTS	Delay of 4.3±2.4 years from ATTR symptom onset to ATTR diagnosis	Biopsy and staining (Abdominal fat, sural nerve) Point mutation testing Point mutations: p.Val30Met, p.Phe64Leu, p.Ala120Ser
Malladi *et al.*, 2019 [62]	Database/registry	562 patients with ATTRv amyloidosis	110 (20%) with CTS	NR	Point mutation testing Point mutations: Val122Ile, Val30Met, and Thr60Ala
Martone *et al.*, 2019 [63]	Case series	70 black patients with ATTRv amyloidosis (V122l point mutation)	64% with CTS	NR	Biopsy and staining Point mutation testing Point mutations: V122I ^99m^PYP/DPD imaging
		19 Caucasian patients with ATTRv amyloidosis (V122l point mutation)	31% with CTS	NR	
Merli *et al.*, 2019 [64]	Case series	11 patients with amyloidosis (8 ATTRwt; 3 types unspecified)	6 with CTS	NR	^99m^PYP/DPD imaging
Milandri *et al.*, 2016 [65]	Case series	109 patients with ATTRv amyloidosis (Glu89Gln point mutation)	>50.0% with CTS	NR	NR
Nakagawa *et al.*, 2016 [66]	Case series	31 patients with ATTRwt amyloidosis	17 (55.0%) with CTS	NR	MSK biopsy and staining (Tenosynovial tissue) Biopsy and staining Gene testing ^99m^PYP/DPD imaging
Ng *et al.*, 2020 [67]	Case series	11 patients with ATTRv amyloidosis (p.Ala117Ser)	3 with CTS	NR	NR
Oike *et al.*, 2021 [68]	Case series	113 patients with ATTRwt amyloidosis	40 (47.0%) with CTS	NR	Biopsy and staining Gene testing ^99m^PYP/DPD imaging
Papoutsidakis *et al.*, 2017 [69]	Case series	17 patients with ATTR amyloidosis (type unspecified)	8 (47.0%) with CTS	NR	^99m^PYP/DPD imaging
Pastorelli *et al.*, 2016 [70]	Case series	20 patients with ATTRwt amyloidosis	15 (75.0%) with CTS	NR	NR
Patel *et al.*, 2021 [71]	Cross-sectional^b^	107 patients with ATTRwt amyloidosis	38% with CTS	NR	LV wall thickening
Peltier *et al.*, 2020 [72]	Case series	9 patients with ATTRv amyloidosis (V142l point mutation)	1 (11.0%) with CTS	NR	Point mutation testing Point mutations: V122I
Peltier *et al.*, 2021 [73]	Case series	18 patients with ATTRv amyloidosis (V142l point mutation)	11 (61.0%) with CTS	NR	Point mutation testing Point mutations: p.V142I ^99m^PYP/DPD imaging
Pinney *et al.*, 2011 [74]	Case series	55 patients with ATTRwt amyloidosis	24 (43.6%) with CTS	7.04(0.54–8.41) survival from symptom onset; 4.58(0.07–5.41) survival from diagnosis	MSK biopsy and staining (carpal tunnel tissue)
Plante-Bordeneuve *et al.*, 2019 [75]	Case series	28 patients with ATTRv amyloidosis (asymptomatic)	5 with CTS (and subsequent CTRS)	NR	MSK biopsy and staining (carpal tunnel nerve) Other biopsy and staining Point mutations: Val30Met ^99m^PYP/DPD imaging
Quarta *et al.*, 2013 [76]	Case series	190 patients with ATTRv amyloidosis (Ile68Leu point mutation)	35.0% with CTS	NR	Gene and/or point mutation testing Point mutations: Ile68Leu
Quarta *et al.*, 2017 [77]	Case series	97 patients with ATTRwt amyloidosis	45 (46.0%) with CTS	NR	LV wall thickening ^99m^PYP/DPD imaging
Ruiz Hueso *et al.*, 2021 [78]	Cross-sectional	13 patients with amyloidosis (84.4% ATTRwt; 7.7% ATTRv; 7.7% non-ATTR)	30.0% with CTS (amyloidosis type not specified)	NR	Gene and/or point mutation testing ^99m^PYP/DPD imaging
Russo *et al.*, 2011 [79]	Case series	23 patients with ATTRv amyloidosis (13 Glu89Gln; 8 Phe64Leu; 2 Thr49Ala)	3 with CTS	NR	Gene and/or point mutation testing Point mutations: Glu89Gln, Phe64Leu, Thr49Ala
Russo *et al.*, 2012 [80]	Case series	18 patients with ATTRv amyloidosis	3 (16.7%) with CTS	NR	LV wall thickening Point mutation testing ^99m^PYP/DPD imaging
Russo *et al.*, 2019 [81]	Database/registry	260 patients with ATTRv amyloidosis	73 with CTS	NR	MSK biopsy and staining Point mutation testing Gene mutations: Phe64Leu, Val30Met, Glu89Gln
Salvalaggio *et al.*, 2021 [82]	Cross-sectional^b^	62 patients with ATTRv amyloidosis	49 (79.0%) with CTS	NR	Gene and/or point mutation testing Point mutations: Phe64Leu, Val30Met, Glu89Gln, Ile68Leu, Thr49Ala, Tyr78Phe, Ala120Ser, Ala36Pro, Arg34Thr, Glu62Lys, Gly47Ala
Salvi *et al.*, 2012 [83]	Database/registry^b^	131 patients with ATTRv amyloidosis	46 (35.1%) with CTS	3(0–13) years delay between clinical ATTRv onset and ATTR diagnosis	Point mutation testing Point mutations: 30 Met, 68 Leu, 34 Thr, 89 Glu, 49 Ala, 34 Thr, 36 Pro, 50 Arg, 47 Arg, 54 Lys, 23 Asn, 53 Ala, 30 Ala, 33 Val, 50 Ser, 14 Leu, 88 Arg, 59 Lys, 54 Gln
Saturi *et al.*, 2020 [84]	Database/registry	4,418 patients with ATTRv amyloidosis	18.6% males with CTS	NR	NR
			15.5% females with CTS	NR	
Shah *et al.*, 2020 [85]	Case series	130 patients with ATTRv amyloidosis	22.3% with CTS	NR	Point mutation testing Point mutations: V142I/V122I
Shah *et al.*, 2021 [86]	Case series	397 patients with ATTRv amyloidosis	24.0% with CTS	NR	Point mutation testing
Shah *et al.*, 2021 [87]	Case series	586 patients with ATTRv amyloidosis	25.0% with CTS	NR	Point mutation testing Point mutations: p.V142I/V122
Silva-Hernández *et al.*, 2020 [88]	Case series	30 patients with ATTRv amyloidosis	15 (20.0%) with CTS	NR	NR
Slama *et al.*, 2021 [89]	Database /registry	4,815 patients with ATTR amyloidosis (type not specified)	18.8% with CTS	NR	NR
Soper *et al.*, 2021 [90]	Database/registry	32 patients with ATTRv amyloidosis (V142l)	10 (31.0%) with CTS	NR	NR
Sousa Paiva *et al.*, 2021 [91]	Case series	30 patients with ATTR amyloidosis (25 ATTRwt; 5 ATTRv [3 Val50Met; 2 Val142lle])	8 (26.7%) with CTS	NR	Biopsy and staining (endomyocardial tissues) Gene and/or point mutation testing Point mutations: Val50Met and Val142lle
Svendsen *et al.*, 1998 [92]	Cross-sectional	25 patients with ATTRv amyloidosis	9 (36.0%) with CTS	NR	MSK biopsy and staining with Congo red (synovial specimen) LV wall thickening Point mutation testing Autopsy
Tzagournissakis *et al.*, 2015 [93]	Case series	17 patients with ATTRv amyloidosis (Met30)	4 with CTS	NR	Point mutation testing Point mutations: Met30
Tzagournissakis *et al.*, 2020 [94]	Case series	10 patients with ATTRv amyloidosis (p.Val114Ala)	10 (100.0%) with CTS	NR	Biopsy and staining Point mutation testing: p.Val114Ala
Warner *et al.*, 2019 [95]	Case series	32 patients with ATTR amyloidosis (type unspecified)	31.0% with CTS	NR	Biopsy and staining LV wall thickening ^99m^PYP/DPD imaging
Yamada *et al.*, 2020 [96]	Cross-sectional	129 patients with ATTRwt amyloidosis	57 (54.0%) with CTS	NR	Biopsy and staining with Congo red (endomyocardial tissues) Gene testing ^99m^PYP/DPD imaging
Yamashita *et al.*, 2020 [97]	Case series	1,937 patients with amyloidosis (13.4% ATTRv; 14.3% ATTRwt; 4.6% ATTR type unspecified)		In 5.6% of total amyloidosis patients (including non-ATTR patients) the initial manifestation of disease was CTS	Point mutations: V30M from an endemic area (7.4%), V30M from a non-endemic area (51.2%), and non-V30M (41.4%)
Zadok *et al.*, 2020 [98]	Case series	26 patients with ATTR amyloidosis (type unspecified)	62.0% with CTS	NR	NR
Zampino *et al.*, 2021 [99]	Case series	56 patients with ATTRv amyloidosis	31 patients with V122l point mutation: 30 (97.0%) with CTS	CTS symptoms preceded ATTR diagnosis by >7years in 30% of patients with V1221	Biopsy and staining with Congo red (skin) Point mutation testing Point mutations: V122I
			12 patients with V30M point mutation: 7 (57.0%) with CTS	CTS symptoms preceded ATTR diagnosis by >7years in 29% of patients with V30M	
			13 patients with L58H point mutation: 10 (77.0%) with CTS	CTS symptoms preceded ATTR diagnosis by >7years in 30% of patients with L58H	
Zivkovic *et al.*, 2020 [100]	Case series	7 patients with ATTRwt amyloidosis	100.0% with CTS	NR	NR

^a^Biopsy and staining of tissues described as other refers to instances where diagnosis was confirmed through staining of non-MSK biopsied tissues, such as endomyocardial tissue

^b^Data from patients with amyloidosis was compared to control patients in these publications

* AS *Aortic stenosis, *ATTR *Transthyretin-mediated amyloidosis, *ATTRv *Hereditary transthyretin amyloidosis, *ATTRwt *Wild-type transthyretin amyloidosis; confidence interval, *CTS *Carpal tunnel syndrome, *DPD *99mTc-3,3-diphosphono-1,2-propanodicarboxylic acid, *LV *Left ventricular, *MS *Mass spectrometry, *MSK *Musculoskeletal, *NR *Not reported, *OR *Odds ratio: PYP = technetium-99m pyrophosphate, *RR *Risk ratio, *SSA *Senile systemic amyloidosis, *TTR *Transthyretin

**Table 3:** ATTR amyloidosis in patients with carpal tunnel syndrome

Publication	Study design	Research population	Prevalence	Additional statistics	Information relating to and/or confirming diagnosis^a^
*ATTR amyloidosis in carpal tunnel syndrome*					
Bäcker *et al.*, 2021 [101]	Case series	699 patients undergoing CTRS	10 (1.4%) with amyloidosis (type unspecified)	NR	MSK biopsy and staining with Congo red (tenosynovium within the carpal tunnel obtained at CTRS)
Bastkjær *et al.*, 2020 [102]	Case series	100 patients with CTS	13 (13.0%) with ATTR amyloidosis (type unspecified)	NR	MSK biopsy and staining with Congo red (Tenosynovial and fatty tissue)
Breda *et al.*, 1993 [103]	Case series	98 patients with CTS	12 (12.2%) with amyloidosis (type unspecified)	NR	MSK biopsy and staining (tenosynovial and flexor retinaculum tissues obtained at CTRS) Staining method NR
Fernandez *et al.*, 2017 [104]	Case series	147 patients with CTS	29 (19.7%) with amyloidosis (type unspecified)	NR	MSK biopsy and staining with Congo red (carpal transverse ligament)
Fosbol *et al.*, 2019 [105]	Database/registry	56,032 patients undergoing CTRS	NR	CTS was associated with a future diagnosis of amyloidosis (type unspecified): HR of 12.2 (95% CI: 4.37–33.60), p <0.0001	NR
Gioeva *et al.*, 2013 [106]	Case series	98 patients who underwent CTS biopsy	98 (100.0%) with ATTR amyloidosis (11 ATTRv, 70 ATTRwt, 17 ATTR unspecified due to lack of genomic DNA available for testing)	NR	MSK biopsy and staining with Congo red (tissues of the transversal carpal ligament) Gene and/or point mutation testing Point mutations: p.G6S & p.M13I
Hahn *et al.*, 2018 [107]	Database/registry	582 patients with CTS	68 (11.7%) with ATTR amyloidosis (type unspecified)	NR	MSK biopsy and staining (tenosynovial and flexor retinaculum tissues obtained at CTRS) Stanning method NR
Hansen *et al.*, 2020 [108]	Case series	182 patients undergoing CTRS	25 (14.0%) with ATTR amyloidosis (type unspecified)	NR	MSK biopsy and staining with Congo red (unspecified tissues obtained at CTRS) Gene and/or point mutation testing _99m_PYP/DPD imaging MS
Itzhaki *et al.*, 2020 [109]	Case series	36 patients with history of CTRS	16 (44.5%) with ATTR amyloidosis (type unspecified)	NR	Biopsy and staining with Congo red (endomyocardial tissues) LV wall thickening Gene and/or point mutation testing ^99m^PYP/DPD imaging
Milandri *et al.*, 2020 [110]	Database/registry	57 patients with history of CTRS	25 (43.9%) with ATTRv amyloidosis and 27 (47.4%) with ATTRwt amyloidosis	Among ATTRv patients, history of CTRS was a strong predictor of later cardiac involvement (positive predictive value 92.0% [95% CI 74.0–99.0%])	LV wall thickening Point mutation testing: Glu89Gln (28.0% of ATTRv), Ile68Leu (48.0% of ATTRv), Val30Met (0.0% of ATTRv), other (24.0%) ^99m^PYP/DPD imaging
				Among ATTRwt patients, history of CTS was associated with an increased risk of death (HR 3.63, [95% CI 1.27–10.3])	
Nakamichi *et al.*, 1996 [111]	Case series	108 patients with a history of CTRS	6 (5.6%) with ATTR amyloidosis (type unspecified)	NR	MSK biopsy and staining with Congo red (tissues of the transversal carpal ligament obtained at CTRS)
Reyes *et al.*, 2017 [112]	Cross-sectional	58 patients undergoing CTRS	5 (8.6%) with ATTR amyloidosis (type unspecified)	NR	MSK biopsy and staining with Congo red (tenosynovial tissues obtained at CTRS) MS ^99m^PYP/DPD imaging
Samões *et al.*, 2017 [113]	Case series	16 patients with history of CTRS	14 (87.5%) with ATTRv amyloidosis	8 (57.1%) patients developed bilateral CTS and were submitted to a second CTRS	MSK biopsy and staining with Congo red (transverse carpal ligaments) Gene and/or point mutation testing Point mutations: V30M
Scott *et al.*, 2019 [114]	Case series	35 patients with a history of CTRS	9 (26.0%) with amyloidosis (7 with ATTRwt amyloidosis; 2 with non-ATTR amyloidosis)	NR	MSK biopsy and staining with Congo red (flexor tenosynovium) MS
Sekijima *et al.*, 2010 [115]	Case series	83 patients with a history of CTRS	28 (35.0%) with ATTRwt amyloidosis	Multivariate logistic regression showed that the prevalence of ATTRwt in the CTS group was significantly high compared to a control group, and age and male gender are independent risk factor for ATTRwt amyloidosis in patients with a history of CTRS.	MSK biopsy and staining with Congo red (tenosynovial tissues obtained at CTRS) Gene and/or point mutation testing MS Autopsy
Sekijima *et al.*, 2011 [116]	Cross-sectional^b^	100 patients with CTS undergoing CTRS	34 (34.0%) with ATTRwt amyloidosis	Binomial logistic regression, corrected for age and sex, showed that ATTRwt amyloidosis in the idiopathic CTS group was significantly higher than that in the control group (odds ratio 15.8, 95% CI 3.29 –75.7)	MSK biopsy and staining with Congo red (tenosynovial tissues obtained at CTRS) Gene testing
Stein *et al.*, 1987 [117]	Case series	140 CTS biopsies	16 (11.4%) with ATTR amyloidosis (type unspecified)	NR	MSK biopsy and staining with Congo red (retinaculum flexor, perineurial fat and connective tissue, and peritendinous and synovial structures)
Sugiura *et al.*, 2021 [118]	s	79 patients with a history of CTRS	27 (34.0%) with ATTR amyloidosis (type unspecified)	16/27 patients with ATTR amyloidosis underwent further testing and all were suspected to have ATTRwt amyloidosis	MSK biopsy and staining with Congo red (tenosynovial tissue within the transverse carpal ligament obtained at CTRS) LV wall thickening ^99m^PYP/DPD imaging
Uchiyama *et al.*, 2014 [119]	Case series	107 patients undergoing CTRS	38 (36.0%) with ATTRwt amyloidosis	NR	MSK biopsy and staining with Congo red (tenosynovial tissues obtained at CTRS) Other biopsy and staining
Vianello *et al.*, 2021 [120]	Cross-sectional	53 male patients with history of CTRS	2 (4.0%) with ATTRwt amyloidosis	NR	LV wall thickening ^99m^PYP/DPD imaging
Wininger *et al.*, 2021 [121]	SLR (case series)^c^	35 patients with CTS	33 (94.2%) with ATTR amyloidosis (type unspecified)	NR	MSK biopsy and staining with Congo red (carpal ligament or synovium)
Zegri-Reiriz *et al.*, 2019 [122]	Cross-sectional	233 patients with history of CTRS	2 (0.9%) with ATTRwt amyloidosis	NR	LV wall thickening ^99m^PYP/DPD imaging

^a^Biopsy and staining of tissues described as other refers to instances where diagnosis was confirmed through staining of non-MSK biopsied tissues, such as endomyocardial tissue

^b^Data from patients with amyloidosis was compared to control patients in these publications

^c^Data from case series investigation by Kyle *et al*. 1992, as reported in the SLR conducted by Wininger *et al.* 2021 on the association between amyloid deposition and MSK pathology

ATTR Transthyretin-mediated amyloidosis, *ATTRv *Hereditary transthyretin amyloidosis, *ATTRwt *Wild-type transthyretin amyloidosis, *CI *Confidence interval, *CTS *Carpal tunnel syndrome, *CTRS *Carpal tunnel release surgery, *DPD *Technetium-99m 3, 3-diphospho-1, 2-propanodicarboxylic acid, *HR *Hazard ratio, *LV *Left ventricular, *MS *Mass spectrometry, *MSK *Musculoskeletal, *NR *Not reported, *OR *Odds ratio, *PYP *Technetium-99m pyrophosphate

**Table 4:** Spinal stenosis in patients with ATTR amyloidosis, and ATTR amyloidosis in patients with spinal stenosis

Publication	Study design	Research population	Prevalence	Additional statistics	Information relating to and/or confirming diagnosis
*Spinal stenosis in ATTR amyloidosis*					
Arevalo *et al.*, 2019 [123]	Database/registry	1,068 patients hospitalized with cardiac amyloidosis (ATTR not specified)	90 (8.4%) with SS	NR	NR
Cortese *et al.*, 2016 [124]	Case series	150 patients with ATTRv amyloidosis	11 (22.0%) patients with previous diagnosis of SS	NR	Biopsy and staining Point mutations: Val30Met (p.Val50Met) Glu89Gln (p.Glu109Gln) Phe64Leu (p.Phe84Leu) Ile68Leu (p.Ile88Leu) Thr49Ala (p.Thr69Ala)
*ATTR amyloidosis in spinal stenosis*					
D'Agostino *et al.*, 1992 [125]	Case series	97 patients with a history of LSS	12 (12.0%) with amyloidosis (type unspecified)	NR	MSK biopsy and staining with Congo red (ligamentum flavum)
Eldhagen *et al.*, 2021 [126]	Cross-sectional	250 patients undergoing LSS	93 (37.0%) with ATTR amyloidosis (type unspecified)	NR	MSK biopsy and staining with Congo red (ligamentum flavum) LV wall thickening
Gagne *et al.*, 1995 [127]	Case series	41 patients with a history of LSS	14 (34.0%) with ATTR amyloidosis (type unspecified)	NR	MSK biopsy and staining with Congo red (ligamentum flavum obtained at LSS)
Gies *et al.*, 1996 [128]	Case series	100 patients with SS	5 (5.0%) with ATTR amyloidosis (type unspecified)	NR	MSK biopsy and staining with Congo red (ligamentum flavum obtained at LSS or surgery for herniated discs)
Godara *et al.*, 2020 [129]	Case series	325 patients with SS	44 (13.0%) with ATTR amyloidosis (type unspecified)	NR	MSK biopsy and staining with Congo red (ligamentum flavum)
Westermark *et al.*, 2014 [130]	Case series	26 patients with history of LSS	5 (19.0%) with ATTR amyloidosis (4 ATTRwt and 1 ATTR type unspecified)	NR	MSK biopsy and staining with Congo red (bone fragments, pieces of ligament and other connective tissue obtained at LSS)
Yanagisawa *et al.*, 2015 [131]	Case series	56 patients with SS	43 (45.3%) with ATTRwt amyloidosis	NR	MSK biopsy and staining with Congo red (ligamentum flavum) LV wall thickening MS

*ATTR *Transthyretin-mediated amyloidosis, *ATTRv *Hereditary transthyretin amyloidosis, *ATTRwt *Wild-type transthyretin amyloidosis, *LSS *Lumbar spinal surgery, *LV *Left ventricular, *MS *Mass spectrometry, *MSK *Musculoskeletal, *NR *Not reported, *SS *Spinal stenosis

**Table 5:** Carpal tunnel syndrome and or spinal stenosis in patients with ATTR amyloidosis

Publication	Study design	Research population	Prevalence CTS	Prevalence SS	Additional statistics	Information relating to and/or confirming diagnosis
*Carpal tunnel syndrome and or spinal stenosis in ATTR amyloidosis*						
Abboud *et al.*, 2020 [132]	Case series	46 patients with cardiac ATTR amyloidosis (type not specified)	10.9% with CTS	21.7% with SS	NR	^99m^PYP/DPD imaging
Arana *et al.*, 2021 [133]	Case series	89 patients with ATTR amyloidosis (83 ATTRwt; 6 ATTRv)	13 (14.8%) with unilateral CTS; 20 (22.7%) with bilateral CTS	21 (23.9%) with SS	NR	Gene and/or point mutation testing Point mutations: Val50Met (100% of ATTRv patients)
Auer-Grumbach *et al.*, 2020 [134]	Case series	22 patients with ATTRv amyloidosis	11 (55.0%) with CTS	2 (1.0%) with SS^a^	NR	Biopsy (endomyocardial tissues) Point mutation testing Point mutations: Val40Ile, Arg41Gln, Val50Met, Thr69Ile, Thr80Ala, His108Arg, Val113Leu, Val114Ala, Ile127Phe, Val142Ile
Aus dem Siepen *et al.*, 2019 [135]	Cross-sectional	77 asymptomatic ATTRv (gene carriers)	10 (13.0%) with CTS	NR	NR	NR
		253 patients with ATTRwt amyloidosis	152 (60.0%) with CTS	35 (14.0%) with SS	32 (12.0%) with CTS and SS	
		136 patients with ATTRv amyloidosis	77 (56.0%) with CTS	7 (5.0%) with SS	3 (2.2%) with CTS and SS	
Bhadola *et al.*, 2020 [136]	Case series	92 patients with ATTRv amyloidosis	73.0% with CTS	18.0% with SS	NR	Point mutation testing Point mutations: V122I, T60A, V30M, L58H, F64L, Y114C, and S77Y
Bukhari *et al.*, 2020 [137]	Case series	440 patients who underwent a ^99m^Tc-PYP skin scan (assumed by authors to be indicative of cardiac ATTR amyloidosis)	NR	NR	OR 4.06 (2.74–5.99), p<0.0001 CTS as a predictor of a positive skin ^99m^PYP test; OR 2.09 (1.39–3.14), p<0.0001 SS as a predictor of a positive skin ^99m^PYP test	^99m^PYP/DPD imaging
Bukhari *et al.*, 2021 [138]	Case series	206 patients with a positive ^99m^Tc-PYP skin scan (assumed by authors to be indicative of cardiac ATTR amyloidosis)	NR	NR	0.48 regression coefficient for bilateral CTS; 0.15 regression coefficient for SS	^99m^PYP/DPD imaging
Campagnolo *et al.*, 2020 [139]	Case series	25 patients with ATTRwt amyloidosis	16 with CTS	2 with SS	NR	Biopsy (endomyocardial and salivary gland tissues) LV wall thickening ^99m^PYP/DPD imaging
Debonnaire *et al.*, 2021 [140]	Case series	114 patients with ATTR amyloidosis (type not specified)	43% with CTS	40% with SS	NR	NR
Di Stefano *et al.*, 2021 [141]	Case series	16 patients with ATTRv amyloidosis	NR	NR	r=0.731 (p=0.0001) association between ATTR and bilateral CTS	NR
			NR	NR	r=0.52 (p=0.040) association between ATTR and SS	
Durmus-Tekçe *et al.*, 2015 [142]	Case series	5 patients with ATTRv amyloidosis (Glu89Gln point mutation)	3 with CTS	1 with SS	NR	Gene and/or point mutation testing Point mutations: Glu89Gln
Durmus-Tekçe *et al.*, 2016 [143]	Case series	17 patients with ATTRv amyloidosis	3 with CTS (all Glu89Gln)	1 with SS (Glu89Gln)	NR	Gene and/or point mutation testing Point mutations: Val30Met, Glu89Gln, Gly53Glu, Glu54Gly, Gly47Glu
Huda *et al.*, 2019 [144]	Database/ registry	373 patients with ATTRwt amyloidosis	NR	NR	CTS as a feature associated with ATTRwt amyloidosis: OR=5.7; 95% CI (4.3–11.8)^b^	NR
					SS as a feature associated with ATTRwt amyloidosis: OR=2.1; 95% CI (1.5–3.1)^b^	
Lauppe *et al.*, 2021 [145]	Database/ registry	994 patients with ATTR cardiac amyloidosis (type not specified)	167 (16.8%) with CTS	86 (8.7%) with SS	NR	NR
Martyn *et al.*, 2021 [146]	Database/ registry	28,825 patients with suspected cardiac ATTR amyloidosis (type not specified)	2,463 (8.5%) with CTS	5,874 (20.0%) with SS	NR	NR
Russo *et al.*, 2020 [147]	Database/ registry	260 patients with ATTRv amyloidosis	21 (8.1%) with CTS	16 (6.2%) with SS	NR	NR
Russell *et al.*, 2021 [148]	Case series	41 patients with ATTRwt amyloidosis	36 (88.0%) with CTS	9 (22%) with SS (6 with history of LSS)	NR	Biopsy (endomyocardial tissues) ^99m^PYP/DPD imaging
*Other*						
George *et al.*, 2020 [149]	Case series	27 patients with ATTRwt amyloidosis and SS	5 (19%) patients had also undergone CTRS	NR	NR	MSK biopsy and staining with Congo red (tissues unspecified) Gene testing MS
George *et al.*, 2021 [150, 151]	Case series	178 patients who underwent LSS with pathology specimens and preoperative MRI	24 (13.5%) with ATTRwt amyloidosis	NR	NR	MSK biopsy and staining (ligamentum flavum obtained from spinal surgery) LV wall thickening MS
		177 patients who underwent LSS with pathology specimens and preoperative MRI	20 (17.0%) with ATTRwt amyloidosis	NR	NR	
		30 patients with surgical indication of SS	6 (20.0%) of patients with ATTRwt amyloidosis+CTS	NR	NR	
		161 patients with surgical indication of SS	4 (16.7%) of patients with ATTRwt amyloidosis+CTS	NR	NR	
Godara *et al.*, 2021 [152]	Cross-sectional	43 patients with ATTR amyloidosis (type not specified) who underwent LSS	15 (35%) with CTS	NR	OR=5.4 (2.2–13.0) CTS independent predictor of ATTR ligamentum flavum deposition	MSK biopsy and staining with Congo red (ligamentum flavum sections) Gene and/or point mutation testing ^99m^PYP/DPD imaging MS

^a^Spinal stenosis was reported in two patients but was not documented and questioned in all patients

^b^findings from a machine learning model of ATTRwt using ICD codes from US medical claims data, compared to a random cohort of HF patients matched by age, gender, and medical histories [cohort 1a (ATTRwt): *N*=373, cohort 1b (HF): *N*=373]

*ATTR *Transthyretin-mediated amyloidosis, *ATTRv *Hereditary transthyretin amyloidosis, *ATTRwt *Wild-type transthyretin amyloidosis, *CTS *Carpal tunnel syndrome, *CTRS *Carpal tunnel release surgery, *DPD *Tc-3,3-diphosphono-1,2-propanodicarboxylic acid, *HF *Heart failure, *ICD *International Classification of Diseases, *IVS *Intraventricular septum, *LSS *Lumbar spinal surgery, *LV *Left ventricular, *MRI *Magnetic resonance imaging, *MS *Mass spectrometry, *NR *Not reported, *OR *Odds ratio, *PYP *Technetium-99m pyrophosphate, *R *Regression, *SLR *Systematic literature review, *SS *Spinal stenosis, *US *United States

**Table 6:** ATTR amyloidosis in patients with osteoarthritis, and ATTR amyloidosis in patients with osteoarthritis

Publications	Study design	Population	Prevalence	Additional statistics	Information relating to and/or confirming diagnosis
*ATTR amyloidosis in osteoarthritis*					
Akasaki *et al.*, 2015 [153–155]	Case series	12 autopsy patients with OA	12 (100%) with amyloid deposits (type unspecified)	NR	MSK biopsy and staining with Congo red (knee cartilage) Autopsy
Egan *et al.*, 1982 [156]	Cross-sectional	18 patients with OA with history of TKA or THA	10 (55.0%) with amyloid deposits (type unspecified)	NR	MSK biopsy and staining with Congo red (cartilage, synovium and articular tissue of the knee and hip) ATTR amyloidosis diagnosis status because of MSK biopsy and staining NR
Gu *et al.*, 2014 [157]	Cross-sectional	36 patients with knee OA and TKA/total knee replacement	8 (22.0%) with amyloid deposits	The mean OA duration in ATTR positive patients was 16.5 (7–30) years compared to ATTR negative patients 12.0 (5–20) years (p=0.014)	MSK biopsy staining with Congo red (synovial specimen obtained at TKA) resulting in a diagnosis of ATTRwt amyloidosis in all patients
Niggemeyer *et al.*, 2011 [158]	Cross-sectional	50 patients with end-stage hip OA who were presently undergoing THA/total hip replacement	17 (33.0%) with amyloid deposits (type unspecified)	NR	MSK biopsy and staining with Congo red (synovium and cartilage of the femoral head obtained at THA) ATTR amyloidosis diagnosis status because of MSK biopsy and staining NR
Takanashi *et al.*, 2013 [159]	Cross-sectional	232 patients with OA and a history of TKA/total knee joint replacement	21 (8.1%) with amyloid deposits (type unspecified)	NR	MSK biopsy and staining with Congo red (synovial tissue obtained at TKA) ATTR amyloidosis diagnosis status because of MSK biopsy and staining NR
Yanagisawa *et al.*, 2016 [160]	Case series	52 patients with OA and a history of TKA	18 (35.3%) with amyloid deposits (type unspecified) in meniscus tissue	NR	MSK biopsy and staining with Congo red (meniscus, articular cartilage, synovial membrane obtained at TKA) ATTR amyloidosis diagnosis status because of MSK biopsy and staining NR
			8 (29.6%) with amyloid deposits (type unspecified) in articular cartilages	NR	
			6 (17.6%) with amyloid deposits (type unspecified) in synovial membrane	NR	
*Osteoarthritis in patients with ATTR amyloidosis*					
Paccagnella *et al.*, 2020 [161]	Database/registry	29 patients with ATTR amyloidosis (20 ATTRwt; 9 unspecified)	59% with THA	NR	NR
			41% with TKA	NR	
Rubin *et al.*, 2017 [162]	Database/registry	156 patients with cardiac ATTR amyloidosis (type unspecified)	20 (12.8%) underwent THA	NR	NR
			22 (14.1%) underwent TKA	NR	

*ATTR *Transthyretin-mediated amyloidosis, *MSK *Musculoskeletal, *NR *Not reported, *OA *Osteoarthritis, *THA *Total hip arthroplasty, *TKA *Total knee arthroplasty

**Table 7:** ATTR amyloidosis in patients where more than one musculoskeletal manifestation was reported and multiple musculoskeletal manifestations in patients with ATTR amyloidosis

Publication	Study design	Population	Prevalence	Additional statistics	Information relating to and/or confirming diagnosis
*Publications where more than one musculoskeletal manifestation in ATTR amyloidosis was reported*					
Campbell *et al.*, 2020 [163]	Case series	36 patients with ATTRwt amyloidosis	52.8% with CTS	NR	Gene and/or point mutation testing: 74% of patients with ATTRv had the p.Val142Ile mutation
			44.4% with CTS with history of CTRS	NR	
			44.4% with SS	NR	
			33.3% with SS with history of LSS	NR	
			ATTRwt: 63.8% with OA with history of JR	NR	
			27.8% with RCI	NR	
		34 patients with ATTRv amyloidosis	ATTRwt: 30.8% with RCI underwent RCR	NR	
			ATTRv: 85.3% with CTS	NR	
			ATTRv: 47.1% with CTS underwent CTS release	NR	
			ATTRv: 41.2% with SS	NR	
			ATTRv: 2.9% with SS underwent laminectomy	NR	
			ATTRv: 17.5% with OA	NR	
Geller *et al.*, 2015 [164]	Case series	99 patients with cardiac ATTR amyloidosis (type unspecified)	30 with CTS	NR	NR
			20 with BTR	NR	
Gorevic *et al.*, 2020 [165]	Case series	31 patients with ATTRv amyloidosis (ATTRIle122 point mutation)	38.7% with CTS	NR	99mPYP scan MS Gene and/or point mutation testing
			25.8% with SS	NR	
			25.8% with OA	NR	
		63 patients with ATTRwt amyloidosis	36.5% with CTS	NR	
			23.8% with SS	NR	
			25.3% with OA	NR	
Kastritis *et al.*, 2020 [166]	Case series	50 patients with ATTRwt amyloidosis	36% with CTS	NR	99mPYP scanning and gene testing
			4% with SS	NR	
			14% with T	NR	
Kogan *et al.*, 2020 [167]	Case series	397 patients with cardiac amyloidosis (70% ATTRwt; 30% ATTRv)	204 (51.4%) with CTS	NR	NR
			101 (25.4%) with SS	NR	
			94 (23.7%) with JR	NR	
			69 (17.4%) with T	NR	
			68 (17.1) with CTS+SS	NR	
			60 (15.1) with CTS+JR	NR	
			49 (12.3) CTS+T	NR	
			35 (8.8) SS+T	NR	
			33 (8.3) SS+JR	NR	
			18 (4.5) T+JR	NR	
			29 (7.3) CTS+SS+T	NR	
			22 (5.5) CTS+SS+JR	NR	
			12 (3.0) CTS+T+JR	NR	
			12 (3.0) SS+T+JR	NR	
Nativi-Nicolau *et al.*, 2020 [168]	Case series	6 patients with ATTRwt amyloidosis	100% with CTS	NR	NR
			32% with SS	NR	
			50% with TF	NR	
Rapezzi *et al.*, 2020 [169]	Case series	106 patients with ATTRv amyloidosis	ATTRv: 27% with CTS	NR	NR
			ATTRv NR with SS	NR	
			ATTRv: 9% with OA	NR	
		335 patients with ATTRwt amyloidosis	ATTRwt: 40% with CTS	NR	
			ATTRwt: 11% with SS	NR	
			ATTRwt: 15% with OA	NR	
Rubin *et al.*, 2017 [170]	Database/registry	Patients with ATTRv amyloidosis	33 (51.6%) with CTS	NR	Point mutation testing
			13 (20.3%) with SS	NR	
			7 (10.9%) with THA	NR	
			5 (7.8%) with TKA	NR	
			3 (4.7%) with RCR	NR	
		Patients with ATTRwt amyloidosis	64 (59.3%) with CTS	NR	NR
			15 (13.9%) with SS	NR	
			15 (13.9%) with THA	NR	
			20 (18.5%) with TKA	NR	
			14 (13.0%) with RCR	NR	
sss Sekijima *et al.*, 2018 [171]	Cross-sectional^a^	51 patients with ATTRwt amyloidosis	10 (20.0%) with CTS as the initial clinical manifestation observed in patients (prior to diagnosis of ATTRwt amyloidosis)	NR	MSK biopsy and staining (tenosynovial tissue within the transverse carpal ligament obtained at CTRS) resulting in a diagnosis of ATTRwt in two patients whose initial clinical manifestation was CTS ATTR amyloidosis diagnosis status because of MSK biopsy and staining NR for the remaining patients Staining method NR
			1 (2.0%) with TF as the initial clinical manifestation observed in patients (prior to diagnosis of ATTRwt amyloidosis )	NR	
			11 (22%) with SS as a clinical manifestation present at diagnosis of ATTRwt amyloidosis	NR	
			23 (45.0%) with CTS as a clinical manifestation present at diagnosis of ATTRwt amyloidosis	NR	
Willis *et al.*, 2021 [172]	Database/registry	1,091 patients “at risk” for ATTRwt amyloidosis	340 (31.0%) with CTS	NR	NR
			654 (60%) with OA		
*Publications where ATTR amyloidosis was reported in more than one musculoskeletal manifestation*					
Hara *et al.*, 2020^b ^ [173]	Case series	20 patients with TF	9 (69.2%) with ATTR amyloidosis (type unspecified)	The mean number of fingers with tenosynovitis was significantly higher in amyloid-positive cases (3.8 fingers) than in amyloid-negative cases (2.0 fingers)^c^	MSK biopsy and staining with the direct fast scarlet method (tendon synovium tissue or flexor tendon sheath tissues obtained at TFRS) resulting in a diagnosis of ATTR (type unspecified) in all patients
Sood *et al.*, 2021 [174]	Database/registry	310 patients with ATTR (type unspecified) following CTRS	122 (39.4%) with bilateral CTS after CTRS	NR	LV wall thickening
			82 (26.5%) with SS after CTRS	NR	
			4 (1.3%) with CTS+BTR	NR	
		89,981 patients with history of CTRS	NR	0.25%, 0.21–0.29% - cumulative incidence of ATTR (type unspecified) after CTRS at 5 years	
			NR	0.55%, 0.47–0.63% - cumulative incidence of ATTR (type unspecified) after CTRS at 10 years	
			NR	0.80%, 0.67–0.93% - cumulative incidence of ATTR (type unspecified) after CTRS at 15 years	
Sperry *et al.*, 2021 [175]	Cross-sectional	13 patients undergoing both TFRS and at least one CTRS	2 (15.4%) with ATTR amyloidosis (type unspecified) in the CTS tenosynovial tissue but not the TF tenosynovial tissue	NR	MSK biopsy and staining with Congo red (tenosynovial tissues obtained at TFRS and CTRS where concomitant CTS was present in patients) ATTR amyloidosis (type unspecified) diagnosis status because of MSK biopsy and staining NR
				NR	
Sperry *et al.*, 2018 [176]	Cross-sectional	98 patients with history of CTRS	1 with ATTR amyloidosis (type unspecified) and CTS	NR	Gene and/or point mutation testing MSK biopsy and staining with Congo red (tenosynovial tissues obtained at CTRS) resulting in a diagnosis of ATTR amyloidosis (type unspecified) in all remaining patients Point mutations: Ala81Thr, Leu58His
			4 with ATTR amyloidosis (type unspecified) and CTS+SS	NR	
			2 with ATTR and CTS+BTR (1 ATTRv and 1 ATTRwt)	NR	
Sueyoshi *et al.*, 2011 [177]	Case series	54 patients with CTS	18 (33.3%) with ATTRwt amyloidosis	NR	MSK biopsy and staining with Congo red (RC tendons, yellow ligaments, and tenosynovial tissues obtained at CTRS) resulting in a diagnosis of ATTRwt amyloidosis in 5 patients (MSK manifestation subgroup not specified)
		21 patients with RCT	5 (23.8%) with ATTRwt amyloidosis	NR	
		36 patients with SS	19 (52.8%) with ATTRwt amyloidosis	NR	

*ATTR *Transthyretin amyloidosis, *ATTRv *Hereditary transthyretin amyloidosis, *ATTRwt *Wild-type transthyretin amyloidosis, BTR; *CTS *Carpal tunnel syndrome, *CTRS *Carpal tunnel release surgery, *JR *Joint replacement, *LSS *Lumbar spinal surgery, *MS *Mass spectrometry, *MSK *Musculoskeletal, *NR *Not reported, *OA *Osteoarthritis, *PYP *Technetium-99m pyrophosphate, *RC *Rotator cuff, *RCI *Rotator cuff injury, *RCR *Rotator cuff repair, *RCT *Rotator cuff tear, *SS *Spinal stenosis, *T *Tendon tear and tendon rupture, *TF *Trigger finger, *TFRS *Trigger finger release surgery, *THA *Total hip arthroplasty, *TKA *Total knee arthroplasty

^a^Data from patients with ATTR amyloidosis was compared to control patients in these publications

^b^For conciseness in reporting, the one publication reporting on an association between TF and ATTR amyloidosis is reported here

^c^This data relates to 13 patients diagnosed with amyloidosis, where 9 were diagnosed with ATTR amyloidosis and the amyloidosis type of the remaining 4 was not specified

**Table 8:** Temporal association between MSK manifestation onset and ATTR amyloidosis diagnosis

Publication	Study design	Population	MSK type and prevalence	Temporal association	Information relating to and/or confirming diagnosis
Auer-Grumbach *et al.*, 2020 [134]	Case series	22 patients with ATTRv amyloidosis	11 (55.0%) with CTS	1–12 years between CTS symptom onset and diagnosis of ATTRv amyloidosis	Biopsy (endomyocardial tissues) Point mutation testing Point mutations: Val40Ile, Arg41Gln, Val50Met, Thr69Ile, Thr80Ala, His108Arg, Val113Leu, Val114Ala, Ile127Phe, Val142Ile
Cappellari *et al.*, 2009 [21]	Case series	14 patients with ATTRv amyloidosis	1 with CTS	Delay from CTS symptom onset to ATTR amyloidosis diagnosis: 2.5 (1–7) years	Biopsy and staining Gene and/or point mutation testing Point mutations: Val30Met, Phe64Leu, Asn124Ser, Glu89Gln, Val122Ile, Ile107Phe, Thr49Ala, Ser50Arg
Cappelli *et al.*, 2021 [23]	Cross-sectional*	168 patients with ATTR amyloidosis (type unspecified)	122 (73%) with CTS	Approximately 8 years between CTS symptom onset and ATTR diagnosis	Point mutation testing: Point mutations: le68leu, Val122ile, other (unspecified)
Debonnaire *et al.*, 2021 [140]	Case series	114 patients with ATTR amyloidosis (type not specified)	43% with CTS 40% with SS	CTS preceded diagnosis with ATTR amyloidosis by 9.5 years; SS preceded diagnosis with ATTR amyloidosis by 7.4 years	NR
Fosbol *et al.*, 2019 [105]	Database/registry	56,032 patients undergoing CTRS	NR	The median time from CTS surgery to diagnosis of ATTR amyloidosis (type unspecified) was 3.1 years	NR
Hewitt *et al.*, 2020 [44]	Case series	15 patients with ATTRv amyloidosis (T60A point mutation)	5 (33%) with CTS	Delay from CTS symptom onset to diagnosis of ATTRv amyloidosis: 2.5(1–7) years	Biopsy and staining Point mutation testing Point mutations: T60a
Itzhaki *et al.*, 2020 [109]	Case series	36 patients with history of CTRS	16 (44.5%) with ATTR amyloidosis (type unspecified)	The median time from CTS diagnosis to diagnosis of ATTR amyloidosis (type unspecified) was 4.3 years	Biopsy and staining with Congo red (endomyocardial tissues) LV wall thickening Gene and/or point mutation testing ^99m^PYP/DPD imaging
Kaku *et al.*, 2020 [47]	Case series	92 patients with ATTRv amyloidosis	72% with CTS	31 patients underwent CTS release, average of 6.7 years from initial CTS symptoms to ATTR diagnosis	NR
Karam *et al.*, 2019 [49]	Case series	23 patients with ATTRv amyloidosis	17 with CTS	Time taken from CTS and diagnosis 11(0–36) years	Point mutation testing Point mutations: Val30Met, Glu89Gln, Gly53Glu, Glu54Gly, Gly47Glu
Kristen *et al.*, 2016 [57]	Case series	191 patients with ATTRwt amyloidosis	87 (48.3%) with CTS	Delay from CTS symptom onset to diagnosis of ATTRwt amyloidosis: 22.2±2.2 months	Gene testing
Leon Cejas *et al.*, 2020 [178]	Case series	94 patients with ATTRv amyloidosis	NR	2 years between SS symptom onset and a diagnosis of ATTRv amyloidosis	Point mutation testing Point mutations: Val30Met (89.4%), Ala97ser (6.4%), Tyr114cys (2.1%), Ile93val (1.1%) and Ala36pro (1.1%)
Rubin *et al.*, 2017 [162]	Database/registry	156 patients with cardiac ATTR amyloidosis (type unspecified)	22 (14.1%) underwent TKA	OA arthroplasty occurred an average of 7.6 years before cardiac ATTR amyloidosis (type unspecified) was diagnosed	NR
Yamada *et al.*, 2020 [96]	Cross-sectional	129 patients with ATTRwt amyloidosis	57 (54.0%) with CTS	Delay from CTS symptom onset to diagnosis of ATTRwt amyloidosis: 15.5 (2–75) months	Biopsy and staining with Congo red (endomyocardial tissues) Gene testing ^99m^PYP/DPD imaging

*AsTTR *Transthyretin amyloidosis, *ATTRv *Hereditary transthyretin amyloidosis, *ATTRwt *Wild-type transthyretin amyloidosis, BTR; *CTS *Carpal tunnel syndrome, *CTRS *Carpal tunnel release surgery, *JR *Joint replacement, *LSS *Lumbar spinal surgery, *MS *Mass spectrometry, *MSK*  Musculoskeletal, *NR *Not reported, *OA *Osteoarthritis, *PYP *Technetium-99m pyrophosphate, *RC *Rotator cuff, *RCI *Rotator cuff injury, *RCR *Rotator cuff repair, *RCT *Rotator cuff tear, *SS *Spinal stenosis, *T *Tendon tear and tendon rupture, *TF *Trigger finger, *TFRS *Trigger finger release surgery, *THA *Total hip arthroplasty, *TKA *Total knee arthroplasty

### Assessment of study quality

A quality assessment of the included publications was performed by one reviewer (and cross-checked by a second to ensure accuracy with discrepancies settled by a third senior reviewer) using the most appropriate Joanna Briggs Institute (JBI) critical appraisal checklist. This assessment was conducted at the publication level [179].

Following the JBI quality assessment of the 163 publications examining the association between MSK manifestations and ATTR amyloidosis, 51 publications were identified as being at low risk of bias [179]. 87 publications had at least one quality domain that implied some potential bias. The most common reason was limited reporting on the method of participant selection and method of diagnosis. In the 25 remaining publications, insufficient information was reported to measure the potential risk of bias.

Of the 13 publications examining the temporal association, four were found to have a low risk of bias, seven had at least one quality domain that implied some potential bias, and in the remaining two publications, there was insufficient information reported to measure the potential risk of bias.

### Data collection and data extraction

The following information from each included publication was extracted: (1) publication characteristics: title, author, publication year, study design, objectives, country, and data collection period, (2) population characteristics: ATTR amyloidosis diagnosis, MSK manifestation subgroup, sample size, and demographic data such as age and sex, (3) the direction of the association relationship (ATTR amyloidosis outcomes in patients with MSK manifestations or MSK manifestations outcomes in patients with ATTR amyloidosis), (4) outcomes as defined in the PICOTS criteria (Supplement 2). Each independent reviewer piloted the data extraction form, and discussions were held to inform any necessary refinements. Data extraction was performed by one reviewer and cross-checked by a second to ensure accuracy. Discrepancies were settled by a third senior reviewer.

## Results

### What evidence supports the association between ATTR amyloidosis and MSK manifestations?

Most studies reported an association between ATTR amyloidosis and CTS (Tables 2 and 3); however, SS, OA, biceps tendon rupture (BTR), rotator cuff injury (RCI), and trigger finger (TF) were also reported and those studies are detailed in Tables 4, 5, 6 and 7. The association between MSK manifestations and the presence of ATTR amyloidosis were reported bi-directionally; for example, some authors investigated CTS in patients with a confirmed diagnosis of ATTR amyloidosis (Table 2), whereas other authors investigated the presence of ATTR amyloidosis in patients who had undergone treatment for CTS (Table 3). When case series were excluded, the prevalence of CTS in patients with ATTR amyloidosis (inclusive of ATTRv and ATTRwt) ranged between 0.5 and 80% (Table 2) [17, 100], and the prevalence of ATTR amyloidosis (inclusive of ATTRv and ATTRwt) in patients with CTS and/or a history of carpal tunnel release (CTR) surgery ranged between 0.9 and 38% (Table 3) [106, 122]. The prevalence of ATTRv amyloidosis in patients with a history of CTR surgery was higher, at 87.5% [113]. Due to the heterogeneity of the studies’ methodologies and approaches, it is not possible to directly compare the prevalences reported. Two publications investigated the prevalence of ATTRv and ATTRwt amyloidoses separately in the same cohort of patients with CTS, finding that ATTRwt amyloidosis was more prevalent in both instances [106, 110].

The prevalence range for SS in patients with ATTR amyloidosis (inclusive of ATTRv and ATTRwt) was narrower than the range reported for CTS, at 8.4–22.0% [123, 124] (Table 4). As observed with CTS, the range of prevalence of ATTR amyloidosis in patients with SS was broader than the range of SS prevalence in patients with ATTR amyloidosis, at 5.0–45.3% (Table 4) [125–131]. Where patients with ATTRwt amyloidosis were the focus, the prevalence of SS ranged between 19.0 and 45.3% [130, 131]. Comparably, Cortese et al. found that in a cohort of patients with ATTRv amyloidosis, 22.0% of patients had previously been diagnosed with SS [124]. In reports where the prevalence of both CTS and SS was explored in the same cohort of patients with ATTR amyloidosis (Table 5), CTS was more prevalent than SS in patients with ATTRv amyloidosis [134–136, 143, 147], as well as in patients with ATTRwt amyloidosis [139].

Several studies investigated ATTR amyloidosis in OA [153–160], and two database/registry studies investigated the presence of OA in patients with ATTR amyloidosis [161, 162] (Table 6). The studies which investigated ATTR amyloidosis in OA explored either the prevalence of amyloid or TTR deposits in patients with OA. In three publications, the presence of amyloid deposits led to a diagnosis of ATTR amyloidosis [157, 158, 160]. For those studies which investigated ATTR amyloidosis in OA, the association between OA and ATTR amyloidosis was confirmed through the staining of biopsy samples taken from the knee and/or hip with Congo red, a standard method used to identify amyloid [153–160]. In patients biopsied during total hip arthroplasty (THA), the prevalence of amyloid deposits in the synovial membrane was 22.0%, leading to a diagnosis of ATTRwt amyloidosis in these patients [157]. In patients biopsied during total knee arthroplasty (TKA), the prevalence of amyloid deposits ranged from 8.1 to 33.0% [158, 159]. In an autopsy study by Akasaki et al., TTR amyloid deposits were present in the knee cartilage and synovial fluid in all 12 autopsies of individuals with OA; no analyses of whether systemic ATTR amyloidosis was present were conducted [153–155]. With respect to the database/registry studies which reported OA in patients with ATTR amyloidosis, the study by Paccagnella et al., reported on 29 patients with ATTR amyloidosis, finding 59% having had THA and 41% having had TKA [161]. The second study by Ruben et al., reported on 156 patients with unspecified ATTR amyloidosis with CM, finding 12.8% having had THA and 14.1% having had TKA [162].

### What is the temporal association between MSK manifestation onset and ATTR amyloidosis diagnosis?

The publications reporting on the temporal association between MSK manifestation onset and a diagnosis of ATTR amyloidosis were limited to CTS, SS, and OA (Fig. 3; Table 8).

Across all CTS-focused publications, CTS symptom onset preceded a diagnosis of ATTR amyloidosis (ATTRv and ATTRwt inclusive) by up to 12 years [21, 23, 44, 47, 49, 57, 96, 105, 109, 134]. In publications reporting on ATTRv amyloidosis separately, the time between CTS symptom onset and diagnosis of ATTRv ranged from 2 to 12 years [21, 44, 47, 49, 134]. This range was 1.3 to 1.9 years in publications reporting on ATTRwt amyloidosis separately [57, 96].

Three studies investigated the temporal association between SS and ATTR amyloidosis; one reported SS symptom onset preceding a diagnosis of ATTRv amyloidosis by approximately 2 years [178], while another reported a 7.4 years delay before an ATTRwt amyloidosis diagnosis [140]. In the same cohort of patients with ATTRwt amyloidosis, CTS symptom onset occurred even earlier than SS symptom onset, preceding the diagnosis of ATTR amyloidosis by 9.5 years [140].

A single publication reported on the temporal association for OA, reporting an average of 7.6 years delay before an ATTR amyloidosis with CM diagnosis was made from OA related surgeries, TKA, and THA [162].

## Discussion

### Background and rationale

The ability to diagnose ATTR amyloidosis early in the disease course is critical to improving patient prognosis, and MSK manifestations may act as an early indicator of ATTR amyloidosis. This systematic review was conducted to investigate the association between ATTR amyloidosis and MSK manifestations, and to investigate the temporal association between MSK manifestation onset and ATTR amyloidosis diagnosis, in order to potentially aid clinicians in identifying and diagnosing the disease earlier. MSK manifestations, including CTS, SS, OA, among others, were found to be associated with a diagnosis of ATTR amyloidosis (Tables 2, 3, 4, 5, 6 and 7). These manifestations were reported to precede the diagnosis of ATTR amyloidosis by years and could be one of the earliest signs of the disease (Table 8). One of the major systemic manifestations of ATTR amyloidosis is CM which causes progressive heart failure, that can lead to significant morbidity and mortality [3, 10]. The number of patients with ATTRv amyloidosis with cardiomyopathy is estimated to be approximately 40,000 to 50,000 globally [10]. Although the exact prevalence of ATTRwt is not known, it is significantly more common than ATTRv, and CM is the most frequent and predominant systemic involvement in ATTRwt amyloidosis [3, 10]. Awareness of and timely detection of MSK manifestations, months or, even years ahead of the beginning of CM can lead to a significant improvement in the care of these patients [180–182].

### Limitations

This systematic review is not all-encompassing, and caution should be exercised when drawing conclusions from such a heterogenous evidence base, including many studies reporting on a small number of patients. With the use of machine learning harnessing big data from registries and electronic health records and advanced statistical methodologies, it may be possible to enhance our understanding of the association between MSK manifestations and ATTR amyloidosis. For example, with the application of machine learning, Willis et al. determined which patients with heart failure were ‘at risk’ for developing ATTR amyloidosis; CTS and OA were highlighted as clinical predictive indicators of interest [172]. The potential benefit of utilizing MSK manifestations associated with ATTR amyloidosis to reduce the delay in diagnosis supports further research in the field.

The included publications were highly heterogenous in terms of how the possible association of ATTR amyloidosis with MSK manifestations was demonstrated (Tables 2, 3, 4, 5, 6 and 7). Biopsy followed by tissue staining of MSK or other specified tissues [14, 16, 18, 21, 22, 25, 27, 30, 33–37, 44, 51, 61, 63, 66, 68, 74, 75, 81, 91, 92, 94–96, 99, 101, 102, 104, 106–115, 117–119, 121, 124–131, 134, 139, 148–150, 152–160, 171, 173, 175, 177, 183] were common. However, detecting amyloid in MSK tissues alone does not necessarily mean a patient is or will be diagnosed with ATTR amyloidosis. Tc-99 m PYP/DPD scintigraphy [19, 20, 24, 29, 37, 39, 41, 42, 45, 51, 58, 60, 63, 64, 66, 68, 69, 73, 77, 78, 80, 95, 96, 109, 110, 118, 120, 122, 132, 137–139, 148, 152, 165, 166], a non-invasive diagnostic method which has been more commonly used during last several years to make a diagnosis of cardiac amyloidosis [3], was also used to confirm the disease in 30% of the publications included in this review (Table 2). Additionally, methods such as mass spectrometry were utilized to confirm that amyloid was caused by TTR [14, 16, 18, 108, 112, 114, 115, 131, 149, 152, 165]. Another significant limitation is that, although an association between MSK manifestations and ATTR amyloidosis is shown in the literature, it does not necessarily demonstrate causation in all cases. Some MSK manifestations seen in patients with (or who will be diagnosed in the future with) ATTR amyloidosis may not be caused by early amyloid deposition. It will be necessary for clinicians and future researchers to take these limitations into account.

### What evidence supports the association between ATTR amyloidosis and MSK manifestations?

The current evidence supports that many MSK manifestations are associated with a diagnosis of ATTR amyloidosis. The MSK manifestation most commonly associated with ATTR amyloidosis is CTS; however, SS, OA, BTR, RCI, TF, among others, were also identified. The exact prevalence of CTS in patients with ATTR amyloidosis remains unclear, with both CTS and ATTR amyloidosis prevalence estimates reported bi-directionally having a broad range. Similarly, no clear trend was identified regarding whether the association with CTS is stronger (indicated by a higher prevalence) in patients with ATTRv or ATTRwt amyloidosis. Nonetheless, given the extent of the identified literature reporting a possible association between CTS and ATTR amyloidosis, patients with CTS may represent a population where targeted screening for ATTR amyloidosis would be valuable [184].

SS was also often associated with ATTR amyloidosis, with similar prevalence estimates identified in patients with ATTRv and ATTRwt amyloidoses. Notably, where the prevalence of CTS and SS was explored in the same patient cohorts with ATTRv or ATTRwt amyloidosis, CTS was more prevalent than SS in all reports [134–136, 139, 143, 147].

Finally, the identified evidence supports that ATTR amyloidosis may be prevalent in patients who previously underwent surgery (THA and/or TKA) for OA. TTR amyloid has been detected in the tissues from the joints of patients with OA, which may or may not be indicative of a diagnosis of ATTR amyloidosis, which was confirmed only in three publications. An interesting case series by Akasaki et al., found that all 12 OA patients who donated their knee articular cartilage for biopsy at autopsy had amyloid deposits in their tissue samples [153–155]. Although further research is needed, the findings of this publication suggest that there may be value for surgeons to consider biopsy and staining with Congo red in patients who undergo knee or hip surgery for OA.

### What is the temporal association between MSK manifestation symptom onset and ATTR amyloidosis diagnosis?

The current evidence highlights that CTS and SS symptom onset can occur months to years, or even decades, before the diagnosis of ATTR amyloidosis [21, 23, 44, 47, 49, 57, 96, 105, 109, 134, 140, 162, 178].

The exact length of time that MSK manifestations precede a diagnosis of ATTR amyloidosis is unclear, with great variation reported across publications. However, the current evidence offers insight into how the temporal association between CTS symptom onset and a diagnosis of ATTR amyloidosis might differ between patients with ATTRv and ATTRwt amyloidosis. According to the current review, CTS symptom onset appears to precede a diagnosis of ATTRv amyloidosis by a substantially longer period than a diagnosis of ATTRwt amyloidosis [21, 44, 47, 49, 96, 134].

Care needs to be taken in the interpretation of the results from these studies given the variation in methodology. For example, at the time of MSK surgery, TTR amyloid deposition may not have occurred within the tissue taken for biopsy, which may confound clinical diagnosis in these patients [153, 160, 175]. Currently, there is no clear order to ATTR amyloid deposition within MSK tissues, i.e., no specific tissue has been identified as the ‘gold standard’ for early detection of ATTR amyloidosis, and biopsy results can vary according to tissue type [153, 160, 175].

## Conclusion

Increased awareness of the MSK manifestations associated with ATTR amyloidosis can enable earlier diagnosis and improve outcomes, given there are effective treatments for this rapidly progressive and fatal condition. Surgeons can play a critical role in early diagnosis of ATTR amyloidosis by recognizing associated MSK manifestations. Currently available data, summarized in this first systematic review conducted on the association between MSK manifestations and ATTR amyloidosis, demonstrates that MSK manifestations can be one of the earliest signs of ATTR amyloidosis; however, it should be kept in mind that the available data is heterogenous, and the extent of the causal relationship between MSK manifestations and ATTR amyloidosis should be further investigated.

### Supplementary Information

**Additional file 1: Supplement 1.** PICOTS criteria for study inclusion and exclusion in the SLR. **Supplement 2.** Ovid® search strategies for EMBASE and Medline (run on November 3rd, 2021). **Supplement 3.** Case studies excluded from the systematic literature with ATTR MSK manifestation

## Data Availability

All data from the review are available within the references included in this manuscript. Only peer-reviewed data reported in published articles, and data presented at congresses and subsequently published as abstracts were used.
