# Supplementary material for: Musculoskeletal manifestations associated with transthyretin-mediated (ATTR) amyloidosis: a systematic review
Source: BMC Musculoskelet Disord. 2023 Sep 22;24:751. doi: 10.1186/s12891-023-06853-5 (PMC10517539; doi:10.1186/s12891-023-06853-5)
Supplement: Supplementary file 1 — Additional file 1: Supplement 1. PICOTS criteria for study inclusion and exclusion in the SLR. Supplement 2. Ovid® search strategies for EMBASE and Medline (run on November 3rd, 2021). Supplement 3. Case studies excluded from the systematic literature with ATTR MSK manifestation [file 12891_2023_6853_MOESM1_ESM.docx]

# Supplementary materials

Supplement 1. PICOTS criteria for study inclusion and exclusion in the SLR

|  | **Inclusion criteria** | **Exclusion criteria** |
| --- | --- | --- |
| **Population** | - Patients with MSK manifestations   AND   - Patients with a diagnosis of ATTR amyloidosis (including detection during autopsy), or patients with a diagnosis of amyloidosis where the subtype is not defined | - Patients with amyloidosis but with no mention of MSK manifestations - Participants with only primary and secondary amyloidosis |
| **Interventions/ comparators** | NA | NA |
| **Outcomes** | - Associations between MSK manifestations and amyloidosis, including but not confined to: - Carpal Tunnel Syndrome - Compression neuropathy - Lumbar spinal stenosis - Bicep brachii muscle - Bicep brachii tendon - Bicep tendon rupture - Tendon rupture - Tendon reconstruction - Rotator cuff injuries - Osteoarthritis - Arthroplasty - Tenosynovitis - Trigger finger - Epidemiology of MSK manifestations in patients with amyloidosis and/or the epidemiology of amyloidosis in patients with MSK manifestations - Incidence - Prevalence - Morbidity - Mortality - The pathophysiology of MSK manifestations associated with amyloidosis - The temporal association between MSK manifestations and amyloidosis - The burden of MSK manifestations associated with amyloidosis - Current clinical practice including: - Yield and location of biopsies - Collaboration between surgeons and amyloid experts - Diagnostic testing for amyloidosis in the context of MSK manifestations - Testing for amyloidosis subtypes in the context of MSK manifestations | - Pathophysiology of MSK manifestations - Efficacy or safety of treatments for amyloidosis or MSK manifestations |
| **Time** | Publications up to 2021 | NA |
| **Study design** | Epidemiological, cross-sectional, retrospective, and prospective cohorts, case control, case reports and SLRs with and without meta-analysis, RCTs | NA |

AA = secondary amyloidosis; AL = primary amyloidosis; ATTR = amyloid transthyretin; MSK = musculoskeletal; NA = not applicable; PICOTS = patient, intervention, comparator, outcome, time, study criteria; RCT = randomized controlled trial; SLR = systematic literature review.

Supplement 2. Ovid® search strategies for EMBASE and Medline (run on November 3rd, 2021)

| **Row** | **Description of terms** | **Query** | **Results** |
| --- | --- | --- | --- |
| *Ovid® search strategy for EMBASE <1988 to 2021 Week 40>* | | | |
| 1 | ATTR and amyloidosis terms | exp ATTR amyloidosis/ or (ATTR or ATTRv or ATTRwt or ATTR PN or ATTR CM or ATTRv PN or ATTRv CM or hATTR or hATTR PN or hATTR CM or wtATTR).mp. or (transthyretin adj3 amyloid*).mp. | 6,473 |
| 2 |  | exp *amyloidosis/ | 27,909 |
| 3 |  | amyloidosis.ti. | 16,801 |
| 4 |  | 1 or 2 or 3  [total amyloidosis terms] | 30,283 |
| 5 | Musculoskeletal and orthopedics terms | exp musculoskeletal disease/ | 2,179,172 |
| 6 |  | exp musculoskeletal diagnosis/ | 388 |
| 7 |  | exp musculoskeletal stress/ | 4,453 |
| 8 |  | exp experimental musculoskeletal disease/ | 7,636 |
| 9 |  | exp musculoskeletal stiffness/ | 1,985 |
| 10 |  | exp experimental musculoskeletal disease/ | 7,636 |
| 11 |  | exp musculoskeletal stiffness/ | 1,985 |
| 12 |  | exp musculoskeletal pain/ | 158,828 |
| 13 |  | exp musculoskeletal disease assessment/ | 61,030 |
| 14 |  | exp orthopedics/ and (exp pain/ or exp injury/) | 8,390 |
| 15 |  | exp arthroplasty/ | 84,825 |
| 16 |  | or/5-15  [total musculoskeletal and orthopedics terms, key words] | 2,227,563 |
| 17 | Specific manifestation terms | exp carpal tunnel syndrome/ | 14,806 |
| 18 |  | exp lumbar spinal stenosis/ | 2,995 |
| 19 |  | exp biceps brachii muscle/ | 10,106 |
| 20 |  | exp tendon rupture/ | 14,907 |
| 21 |  | exp tendon reconstruction/ | 8,658 |
| 22 |  | exp rotator cuff rupture/ | 7,837 |
| 23 |  | exp osteoarthritis/ | 132,517 |
| 24 |  | exp tenosynovitis/ | 6,741 |
| 25 |  | exp trigger finger/ | 924 |
| 26 |  | or/17-25  [total manifestation terms] | 183,309 |
| 27 | Musculoskeletal, orthopedic and manifestation terms | (musculoskel* adj3 (rupture* or tear or reconstruct* or surgical or surger* or disease or function or manifest* or damage or trauma)).mp. | 45,256 |
| 28 |  | (ligament* adj3 (rupture* or tear or reconstruct* or surgical or surger* or disease or function or manifest* or damage or trauma)).mp. | 32,975 |
| 29 |  | (bicep* adj3 (rupture* or tear or reconstruct* or surgical or surger* or disease or function or manifest* or damage or trauma)).mp. | 1,163 |
| 30 |  | ((joint* or hip* or knee*) adj3 (replace* or arthroplasty* or surgical or surger*)).mp. | 134,856 |
| 31 |  | ((tendon* or rotator cuff*) adj3 (rupture* or tear or reconstruct*)).mp. | 25,185 |
| 32 |  | (trigger finger adj3 (release or surger* or surgical)).mp. | 204 |
| 33 |  | (orthop$edic* adj3 (rupture* or tear or reconstruct* or surgical or surger* or disease or function or manifest* or damage or trauma)).mp. | 46,815 |
| 34 |  | carpal tunnel.mp. | 16,599 |
| 35 |  | CTS.mp. | 17,399 |
| 36 |  | arthroscop*.mp. | 51,782 |
| 37 |  | osteoarthritis.mp. | 148,189 |
| 38 |  | tenosynovitis.mp. | 6,713 |
| 39 |  | (median nerve adj3 entrapment).mp. | 297 |
| 40 |  | spinal stenosis.mp. | 8,668 |
| 41 |  | or/27-40  [total musculoskeletal and orthopedics terms, free text terms] | 436,905 |
| 42 |  | 16 or 26 or 41  [total musculoskeletal manifestations] | 2,324,843 |
| 43 |  | 4 and 42  [total with revised amyloidosis and musculoskeletal] | 5,147 |
| 44 |  | ((exp animal/ or nonhuman/) not human/) or exp animal experiment/ or exp animal model/ or exp rodent/ or (rat or rats or mouse or mice).ti. | 6,392,192 |
| 45 |  | Letter/ or editorial/ or (letter or comment*).ti. | 1,758,565 |
| 46 |  | 43 not (44 or 45)  [removing animal studies and any letters or editorials from the most focused search] | 4,518 |
| *Ovid® search strategy for Medline* | | | |
| 1 | ATTR and amyloidosis terms | exp Amyloidosis/ or exp Amyloid Neuropathies, Familial/ | 26,140 |
| 2 |  | (ATTR or ATTRv or ATTRwt or ATTR PN or ATTR CM or ATTRv PN or ATTRv CM or hATTR or hATTR PN or hATTR CM or wtATTR or (transthyretin adj3 amyloid*)).mp. | 3,023 |
| 3 |  | amyloidosis.ti. | 15,806 |
| 4 |  | or/1-3 | 28,382 |
| 5 | Musculoskeletal and orthopedic terms | exp Musculoskeletal Abnormalities/ or exp Musculoskeletal pain/ or exp Musculoskeletal Diseases/ or exp Carpal Tunnel Syndrome/ or exp Lumbar Vertebrae/ or exp Tendon Injuries/ or exp Rupture/ or exp Rotator Cuff Injuries/ or exp Osteoarthritis/ or exp Tenosynovitis/ or exp Trigger Finger Disorder/ or exp Spinal Stenosis/ | 1,223,007 |
| 6 |  | exp Orthopedics/ | 22,347 |
| 7 |  | exp Pain/ | 422,689 |
| 8 |  | exp "Wounds and Injuries"/ | 953,888 |
| 9 |  | exp Arthroplasty/ | 78,807 |
| 10 |  | (musculoskel* adj3 (rupture* or tear or reconstruct* or surgical or surger* or disease or function or manifest* or damage or trauma)).mp. | 4,642 |
| 11 |  | (ligament* adj3 (rupture* or tear or reconstruct* or surgical or surger* or disease or function or manifest* or damage or trauma)).mp. | 21,276 |
| 12 |  | (bicep* adj3 (rupture* or tear or reconstruct* or surgical or surger* or disease or function or manifest* or damage or trauma)).mp. | 983 |
| 13 |  | ((joint* or hip* or knee*) adj3 (replace* or arthroplasty* or surgical or surger*)).mp. | 101,700 |
| 14 |  | ((tendon* or rotator cuff*) adj3 (rupture* or tear or reconstruct*)).mp. | 13,247 |
| 15 |  | (trigger finger adj3 (release or surger* or surgical)).mp. | 208 |
| 16 |  | (orthop$edic* adj3 (rupture* or tear or reconstruct* or surgical or surger* or disease or function or manifest* or damage or trauma)).mp. | 14,424 |
| 17 |  | (carpal tunnel or CTS).mp. | 19,890 |
| 18 |  | osteoarthritis.mp. | 98,584 |
| 19 |  | tenosynovitis.mp. | 4,729 |
| 20 |  | (median nerve adj3 entrapment).mp. | 243 |
| 21 |  | spinal stenosis.mp. | 9,079 |
| 22 |  | arthroscop*.mp. | 40,196 |
| 23 |  | or/5-22 | 1,366,984 |
| 24 |  | 4 and 23 | 2,900 |
| 25 |  | ((exp animal/ or nonhuman/) not human/) or exp animal experiment/ or exp animal model/ or exp rodent/ or (rat or rats or mouse or mice).ti. | 5,893,141 |
| 26 |  | 24 not 25 | 2,801 |
| 27 |  | Letter/ or editorial/ or (letter or comment*).ti. | 1,846,374 |
| 28 |  | 26 not 27 | 2,570 |

Supplement 3. Case studies excluded from the systematic literature with ATTR MSK manifestation

| **Publications** | **MSK manifestation** |
| --- | --- |
| Ahmed TY, A. Lodhi, S. H. Hanna, M. Chung, R. Left Bundle Branch Pacing in Cardiac Amyloidosis. *J Am Coll Cardiol.* 2020;75(11):2346. | CTS |
| Akin RKB, K. Walters, P. J. Amyloidosis, macroglossia, and carpal tunnel syndrome associated with myeloma. *J Oral Surg.* 1975;33:690-692. | CTS |
| Akinlonu AG, A. N. Mene-Afejuku, T. Akinboboye, O. Transthyretin Cardiac Amyloidosis Masquerading as Hypertensive Cardiomyopathy with Recurrent Decompensation. *J Am Coll Cardiol.* 2021;77(18 Supplement 1):2942. | CTRS |
| Alexander Valerievitch Nossikoff AK, N. Lazarova, G. Slavchev, B. Donova, T. Direct confirmation of suspected hereditary ATTR by genetic sequencing. *Eur J Heart Fail.* 2019;21(Supplement 1):501-502. | CTS |
| Authier FJL-Z, E. Mussini, J. M. Plante-Bordeneuve, V. Eizenbaum, J. F. Jacobson, D. R. Gherardi, R. K. Marked systemic amyloid angiopathy in patients with val 107 transthyretin mutation. *J.* 1999;1:82-85. | CTS |
| Barata Silverio TC, J. Mendes, I. Taipa, R. Melo Pires, M. Pereira, P. Late-onset Familial Amyloid Polyneuropathy associated with transthyretin: Diagnostic challenge. *Eur J Neurol.* 2019;26(Supplement 1):537. | CTS |
| Benson MD, 2nd Julien, J. Liepnieks, J. Zeldenrust, S. Benson, M. D. A transthyretin variant (alanine 49) associated with familial amyloidotic polyneuropathy in a French family. *J Med Genet.* 1993;30:117-119. | CTS |
| Benson MDK, J. C. Transthyretin GLY67ARG: Peripheral neuropathy and vitreous amyloidosis. *J Peripher Nerv Syst.* 2013;2):S13. | CTS |
| Bertoia BM, M. G. Tinarelli, G. [Acquired systemic amyloidosis associated with monoclonal gammopathy]. *Minerva Med.* 1985;76:1289-1292. | CTS |
| Blanco-Jerez CRJ-E, A. Gobernado, J. M. Lopez-Calvo, S. de Blas, G. Redondo, C. Garcia Villanueva, M. Orensanz, L. Transthyretin Tyr77 familial amyloid polyneuropathy: a clinicopathological study of a large kindred. *Muscle Nerve.* 1998;21:1478-1485. | CTS |
| Boyd AH, A. Zenker, M. Fatal Attraction. *Chest.* 2021;160(4 Supplement):A177-A178. | CTS |
| Boyle RPS, J. Schwartz, G. Carpal Tunnel Syndrome in Transthyretin Cardiac Amyloidosis: Implications and Protocol for Diagnosis and Treatment. *Cureus.* 2021;13:e14546. | CTS |
| Briani CT, F. Cavallaro, T. Ferrari, S. Calamelli, S. Adami, F. Fabrizi, G. M. Transthyretin amyloidosis (ATTR) due to a newttr gene mutation, misdiagnosed as primary amyloidosis. *J Peripher Nerv Syst.* 2011;3):S13. | CTS |
| Cappellari MF, M. Taioli, F. Cavallaro, T. Ferrari, S. Rizzuto, N. Fabrizi, G. M. Carpal tunnel syndrome: A possible long-standing heralding sign of transthyretinopathy? *J Peripher Nerv Syst.* 2009;1):5-6. | CTS |
| Carr ASS, S. Choi, D. Blake, J. Phadke, R. Gilbertson, J. Whelan, C. J. Wechalekar, A. D. Gillmore, J. D. Hawkins, P. N. Reilly, M. M. Spinal Stenosis in Familial Transthyretin Amyloidosis. *J Neuromuscul Dis.* 2019;6:267-270. | CTRS |
| Carstens PHW, D. Crystalline glomerular inclusions in multiple myeloma. *Am J Kidney Dis.* 1989;14:56-60. | CTS |
| Cash TG, N. Mozaffar, T. TTR familial amyloid polyneuropathy presenting with tongue atrophy and fasciculations. *J.* 2014;1):8-9. | CTRS |
| Chapman RHC, F. The carpal tunnel sydrome and amyloidosis. A case report. *Clinical Orthopaedics and Related Research.* 1982;169:159-162. | CTS |
| Chen BZ, Z. Niu, S. Tai, H. Pan, H. Dong, G. Qu, Y. Multiple cranial nerves were damaged in a patient with familial amyloidosis Finnish type. [Chinese]. *Chinese Journal of Neurology.* 2021;54(6):579-584. | CTS |
| Civera SDL, A. Collevecchio, A. Previtero, M. Vio, R. Angelini, A. Fedrigo, M. Perazzolo Marra, M. Badano, L. Briani, C. Cecchin, D. Cacciavillani, M. Berno, T. Iliceto, S. Calore, C. An "arrhythmic" case of cardiac amyloidosis. *J Peripher Nerv Syst.* 2019;24(Supplement 1):S12. | CTS |
| Civera SDL, A. Collevecchio, A. Previtero, M. Vio, R. Angelini, A. Fedrigo, M. Perazzolo Marra, M. Badano, L. Briani, C. Cecchin, D. Cacciavillani, M. Berno, T. Iliceto, S. Calore, C. Multidisciplinary approach to cardiac transthyretin-related amyloidosis: A case series. *J Peripher Nerv Syst.* 2019;24(Supplement 1):S13. | CTRS |
| Dalla Torre CT, F. Lucchetta, M. Baracchini, C. Cagnin, A. Fabrizi, G. M. Briani, C. Delayed diagnosis of oculoleptomeningeal amyloidosis with a tyr114cys mutation. *J Peripher Nerv Syst.* 2013;1):S11. | CTS |
| Darwich CG, E. Berk, J. L. Zigante, N. Diagnosis and Management of a Sibling Pair with Hereditary Transthyretin Amyloidosis Associated with F44S (p.Phe64Ser) Transthyretin Variant: A Case Report. *Neurology. Conference: 73rd Annual Meeting of the American Academy of Neurology, AAN.* 2021;96. | CTS |
| Davies TS, A. Coghlan, G. Whelan, C. Agarwal, B. A case study of likely wild-type cardiac transthyretin amyloidosis causing rapid deterioration. *J.* 2017;18:138-142. | CTRS |
| De Bruijn SG, X. Verhelst, J. Prihadi, E. A special case of isolated cardiac amyloidosis. *Acta Clinica Belgica: International Journal of Clinical and Laboratory Medicine.* 2018;73(Supplement 2):22-23. | CTS |
| Dematte CG, C. Vinci, A. Viliani, D. Gambaro, A. Caruso, F. Marinetti, A. Della Sala, S. Del Greco, M. Inverse multimodality diagnostic process in a case of transthyretin cardiac amyloidosis and prostate cancer. *European Heart Journal, Supplement.* 2020;22(SUPPL G):G167. | CTS |
| Dowd RSN, T. J. Arkun, K. Kryzanski, J. Soto, O. Fogaren, T. Harrington, K. Patel, A. Comenzo, R. Riesenburger, R. I. Where Neurosurgery Meets Heart Failure: A Case Report of a Patient with Amyloid Transthyretin Wild Type in the Ligamentum Flavum and Cardiac Tissue with Bilateral Carpal Tunnel Syndrome. *World Neurosurg.* 2019;131:104-107. | CTS |
| Endicott JNC, J. S. Amyloidosis presenting as a mass in the neck. *Laryngoscope.* 1979;89:1224-1228. | CTS |
| Fattorello Salimbeni CT, F. Cabrini, I. Cappellari, M. Serena, M. Cavallaro, T. Fabrizi, G. M. Co-occurrence of TTR and MPZ mutations in late-onset peroneal atrophy: A diagnostic puzzle. *J Peripher Nerv Syst.* 2011;2):S15. | CTS |
| Ferrara MAM, S. Ultrasound examination of the wrist. *J Belge Radiol.* 1997;80:78-80. | CTS |
| Geraldes TMP, M. Coelho, T. A difficult differential diagnosis in a case of small fiber neuropathy. *Eur J Neurol.* 2015;1):676. | CTS |
| Giglio GA, G. Carrozza, F. Obici, L. Musacchio, M. Hereditary amyloidosis by transthyretin: Diagnosis of heterozygous mutation Val30Met in two brothers. *Amyloid.* 2010;1):134-135. | CTS |
| Goh KJK, J. H. Kim, B. J. Tan, C. T. Familial transthyretin-related amyloid polyneuropathy in a Malaysian patient of ethnic Chinese descent. *Neurology Asia.* 2008;13(2):121-124. | CTS |
| Griffee SR, R. K. Bilateral brachial plexopathy: Differential diagnosis of amyloidosis? A case report. *PM and R.* 2013;1):S159. | CTS |
| Hakyemez OSA, F. Birinci, M. Cacan, M. A. Kara, A. Acute Carpal Tunnel Syndrome Secondary to Amyloidosis. *Case Rep Orthop.* 2019;2019:1610430. | CTS |
| Hallett J. Tendon tethering in the carpal tunnel due to amyloidosis in Bence-Jones myelomatosis. *J Bone Joint Surg Br.* 1982;64:357-360. | CTS |
| Hamour IML, H. J. Goodman, H. J. B. Petrou, M. Burke, M. M. Hawkins, P. N. Banner, N. R. Heart transplantation for homozygous familial transthyretin (TTR) V122I cardiac amyloidosis. *American Journal of Transplantation.* 2008;8(5):1056-1059. | CTS |
| Hernandez Jimenez SAS, G. Rajjoub Al-Mahdi, E. Sanchez Vega, J. D. Ramos Jimenez, J. Plaza Martin, M. Zamorano Gomez, J. L. A heart failure with brilliantly preserved ejection fraction. *European Heart Journal Cardiovascular Imaging.* 2020;21(Supplement 1):i552. | CTS |
| Holmgren GH, U. Jonasson, J. Lundgren, H. E. Westermark, P. Suhr, O. B. A Swedish family with the rare Phe33Leu transthyretin mutation. *Amyloid.* 2005;12:189-192. | CTS |
| Ishiyama TS, N. Miyayama, T. Sugimoto, M. Wakabayashi, Y. Shiokawa, Y. [A case of multiple myeloma complicated with amyloidosis and carpal tunnel syndrome]. *Rinsho Ketsueki.* 1984;25:1809-1813. | CTS |
| Janunger TA, I. Holmgren, G. Lovheim, O. Ohlsson, P. I. Suhr, O. B. Tashima, K. Heart failure caused by a novel amyloidogenic mutation of the transthyretin gene: ATTR Ala45Ser. *Amyloid.* 2000;7:137-140. | CTS |
| Jayakrishnan TK, A. Shah, D. Guha, A. Salman Faisal, M. Mewawalla, P. Senile Systemic Amyloidosis Presenting as Hematuria: A Rare Presentation and Review of Literature. *Case Report Med.* 2020;2020:5892707. | CTS |
| Kanoh TY, K. Ohnaka, T. Rapid progression of systemic amyloidosis after high-dose corticosteroid therapy in multiple myeloma. [Japanese]. *[Rinsho ketsueki] The Japanese journal of clinical hematology.* 1990;31(10):1736-1739. | CTS |
| Khella SD, B. DiVito, P. Brannigan, T. Polydefkis, M. Neurologic involvement in V122i familial amyloidosis. *Ann Neurol.* 2016;80(Supplement 20):S222. | CTS |
| Khoo HWD, C. S. L. Tandon, A. A. Radiologic Findings in Polyarticular Amyloid Arthropathy and Myopathy in Multiple Myeloma: A Case Report. *Am J Case Rep.* 2018;19:1398-1404 | CTS |
| Klaassen SHCL, H. H. Bijzet, J. Glaudemans, Awjm Bos, R. Plattel, W. van den Berg, M. P. Slart, Rhja Nienhuis, H. L. A. van Veldhuisen, D. J. Hazenberg, B. P. C. Late onset cardiomyopathy as presenting sign of ATTR A45G amyloidosis caused by a novel TTR mutation (p.A65G). *Cardiovasc Pathol.* 2017;29:19-22. | CTS |
| Koyama SK, T. Kurokawa, K. Tanji, H. Iseki, C. Arawaka, S. Wada, M. Kato, T. Carpal tunnel syndrome as an initial manifestation in a case of transthyretin-related familial amyloid polyneuropathy with a novel A120T mutation. *Clin Neurol Neurosurg.* 2012;114:707-709. | CTS |
| Kuntzer TO, F. What to do when the neuropathy worsens after successful heart and liver transplantation in a Glu89Lys transthyretin amyloidosis? *Orphanet Journal of Rare Diseases. Conference: 1st European Congress on Hereditary ATTR Amyloidosis. Paris France.* 2015;10. | CTS |
| Kuzume DS, K. Morimoto, Y. Komatsu, K. Yamasaki, M. Enzan, H. A case of familial amyloid polyneuropathy (FAP ATTR Ile107Val) with proximal muscle weakness in the lower extremities. *Rinsho Shinkeigaku.* 2016;56:277-280. | CTS |
| Levy JH, P. N. Rowczenio, D. Godfrey, T. Stawell, R. Zamir, E. Familial amyloid polyneuropathy associated with the novel transthyretin variant Arg34Gly. *Amyloid.* 2012;19:201-203. | CTS |
| Lim K. Application of a new noninasive diagnostic approach to transthyretin cardiac amyloidosis. *J Gen Intern Med.* 2019;34(2 Supplement):S486. | CTS |
| Loh FCR, N. Yeo, J. F. Amyloidosis with oral involvement. Case report. *Aust Dent J.* 1990;35:14-18. | CTS |
| Lossos AS, D. Steiner-Birmanns, B. Hassin-Baer, S. Sadeh, M. Sagi, M. Linetski, E. Abramsky, O. Argov, Z. Rosenmann, H. Extended phenotype in the transthyretin Tyr77 familial amyloid polyneuropathy. *Eur Neurol.* 2005;53:55-59. | CTS |
| Magy-Bertrand NB, M. D. Meaux-Ruault, N. Valleix, S. Gil, H. Elastorrhexia, macroglossia and enlarging salivary glands: Three new clinical features of the Y78F transthyretin mutation. *Amyloid.* 2010;1):140. | CTS |
| Magy NL, J. J. Gil, H. Kantelip, B. Dupond, J. L. Kluve-Beckerman, B. Benson, M. D. A transthyretin mutation (Tyr78Phe) associated with peripheral neuropathy, carpal tunnel syndrome and skin amyloidosis. *Amyloid.* 2003;10:29-33. | CTS |
| Mahuwala ZE, J. Clinical presentation of a patient with late onset TTR related amyloidosis. *J.* 2016;17(3):162-163. | CTS |
| Martini NR, S. Sarais, C. Cipriani, A. Negative bone scintigraphy in wild-type transthyretin cardiac amyloidosis. *BMC Cardiovasc Disord.* 2020;20:466 | CTS |
| Michael A. Restrictive cardiomyopathy due to cardiac amyloidosis. *European Geriatric Medicine.* 2020;11(SUPPL 1):S149. | CTS |
| Misrahi AMP, V. Lalu, T. Serre, L. Adams, D. Lacroix, D. C. Said, G. New transthyretin variants SER 91 and SER 116 associated with familial amyloidotic polyneuropathy. Mutations in brief no. 151. Online. *Hum Mutat.* 1998;12:71. | CTS |
| Mochizuki HK, K. Masaki, T. Hirata, A. Tokuda, T. Yazaki, M. Motoyoshi, K. Ikeda, S. Nodular cutaneous amyloidosis and carpal tunnel syndrome due to the amyloidogenic transthyretin His 114 variant. *Amyloid.* 2001;8:105-110. | CTS |
| Mohr W. Granulomatous amyloidosis - A characteristic substrate of transthyretin amyloidosis in carpal tunnel syndrome. [German]. *Aktuelle Rheumatologie.* 2001;26(1):26-29. | CTS |
| Morgado GJG, A. C. Cruz, I. R. Carmona, S. Fazendas, P. Joao, I. Santos, A. I. Lopes, L. R. Pereira, H. Sometimes it is more than just coronary atherosclerosis. *European Heart Journal Cardiovascular Imaging.* 2016;17(Supplement 2):ii183. | CTS |
| Motozaki YS, Y. Ishida, C. Komai, K. Matsubara, S. Yamada, M. Phenotypic heterogeneity in a family with FAP due to a TTR Leu58Arg mutation: a clinicopathologic study. *J Neurol Sci.* 2007;260:236-239. | CTS |
| Mouksian KR, A. Yedlapati, N. Pullen, D. Jefferies, J. Amyloidosis - a Novel TTR Mutation Found in an Asian Female. *J Am Coll Cardiol.* 2021;77(18 Supplement 1):1959. | CTS |
| Mufuka BA, K. Craig, M. A common presentation of the uncommon. *Journal of Hospital Medicine. Conference: Hospital Medicine, HM.* 2018;13. | CTS |
| Murakami TT, S. Endo, Y. Kawai, R. Hara, M. Tanase, S. Ando, M. Familial carpal tunnel syndrome due to amyloidogenic transthyretin His 114 variant. *Neurology.* 1994;44:315-318. | CTS |
| Nakase TY, T. Matsuo, Y. Nomura, T. Sasada, K. Masuda, T. Misumi, Y. Takamatsu, K. Oda, S. Furukawa, Y. Obayashi, K. Matsui, H. Ando, Y. Ueda, M. Hereditary ATTR Amyloidosis with Cardiomyopathy Caused by the Novel Variant Transthyretin Y114S (p.Y134S). *Intern Med.* 2019;58:2695-2698. | CTS |
| Nanri KU, H. Yamada, M. Takata, Y. Matsumura, A. Kougo, K. Sekine, S. Ogawa, D. Toyoda, M. Transthyretin Val 107 in a Japanese patient with familial amyloid polyneuropathy. *J Neurol Sci.* 2002;198:93-96. | CTS |
| Nestle FOB, G. Bilateral carpal tunnel syndrome as a clue for the diagnosis of systemic amyloidosis. *Dermatology.* 2001;202:353-355. | CTS |
| Nicula AR, R. Marinescu, A. Vinereanu, D. Iana, G. Cardiac amyloidosis - The importance of mapping sequences. *European Heart Journal Cardiovascular Imaging.* 2019;20(Supplement 2):ii284. | CTS |
| Noto YT, T. Shiga, K. Tsuchiya, A. Yazaki, M. Matoba, S. Nakagawa, M. Cardiomyopathy in a Japanese family with the Glu61Lys transthyretin variant: a new phenotype. *Amyloid.* 2009;16:99-102. | CTS |
| Obici LP, S. Mussinelli, R. Arbustini, E. Tasaki, M. Lavatelli, F. Casarini, S. Raimondi, A. Merlini, G. The relationship between 99mTc-DPD uptake and amyloid fibril composition in hereditary cardiac TTR amyloidosis: Is the Glu92Lys variant an exception to the rule? *Orphanet Journal of Rare Diseases. Conference: 1st European Meeting for ATTR Amyloidosis for Doctors and Patients. Paris France.* 2017;12. | CTS |
| Ohya YT, M. Hayashida, S. Katayama, N. Tsuchida, T. Kuriwaki, K. Ueda, M. Inomata, Y. Carpal Tunnel Syndrome Due to Iatrogenic Amyloidosis After Domino Liver Transplantation From Hereditary Transthyretin Amyloidosis: A Case Report. *Transplant Proc.* 2021;53:1313-1316. | CTS |
| Pedraza MIG, A. L. Fernandez, N. Telleria, J. J. Ruiz Pinero, M. Galan, L. Familial amyloid polyneuropathy with Ser97Tyr transthyretin mutation: 2 new cases. *Eur J Neurol.* 2016;2):472. | CTS |
| Pelter MA, M. Mohan, R. Hands Stained with Congo Red: Diagnosing ATTR Amyloid in a Patient with Longstanding Heart Failure with Reduced Ejection Fraction and New Carpal Tunnel Syndrome. *J Am Coll Cardiol.* 2021;77(18 Supplement 1):2189. | CTS |
| Pradotto LM, M. Vigna, L. Cattaldo, S. Di Blasio, A. Mauro, A. Expanding the clinical features of the Glu51Gly TTR gene mutation. *Clin Neuropathol.* 2018;37(3):135. | CTS |
| Pradotto LO, L. Milesi, A. Mura, E. Sellitti, L. Mauro, A. Bilateral carpal tunnel syndrome and vagal paroxysmal atrial fibrillation as clinical presentation of Glu89Gln TTR mutation. *Clin Neuropathol.* 2013;32(3):235. | CTS |
| Raivio VEJ, J. Myllykangas, L. Ala-Mello, S. Kankuri-Tammilehto, M. Kiuru-Enari, S. Westermark, P. Tanskanen, M. Kivela, T. A novel transthyretin Lys70Glu (p.Lys90Glu) mutation presenting with vitreous amyloidosis and carpal tunnel syndrome. *Amyloid.* 2016;23:46-50. | CTS |
| Riboldi GDB, R. Ranieri, M. Magri, F. Sciacco, M. Moggio, M. Bresolin, N. Corti, S. Comi, G. P. Tyr78Phe Transthyretin Mutation with Predominant Motor Neuropathy as the Initial Presentation. *Case Rep Neurol.* 2011;3:62-68. | CTS |
| Rittmeyer KS, J. [Paramyloidosis, macroglossia and carpal tummel syndrome in plasmacytoma]. *Dtsch Med Wochenschr.* 1968;93:353-355. | CTS |
| Robert-Daniel Adam RDJ, A. Badelita, S. Fruntelata, A. G. Ciudin, R. Popescu, B. A. Ginghina, C. Draghici, M. Stan, C. Coriu, D. Jurcut, R. Heart failure aggravated by beta blockers. Could this suggest the etiology? *Eur J Heart Fail.* 2019;21(Supplement 1):591 | CTS |
| Rodriguez AL, P. Pedelhez, R. Ladys, M. Rare mutational variant (Ile93Val) of heredity amyloidosis with severe dysautonomia treated with inotersen (ASO). *J Peripher Nerv Syst.* 2020;25(4):461. | CTS |
| Rosenzweig MS, M. Prokaeva, T. Theberge, R. Costello, C. Drachman, B. M. Connors, L. H. A new transthyretin variant (Glu61Gly) associated with cardiomyopathy. *Amyloid.* 2007;14:65-71. | CTS |
| Rubio Alonso BJR, A. Molina Martin De Nicolas, J. Diaz Anton, B. Barrios Garrido- Lestache, E. Martin Asenjo, R. Delgado Jimenez, J. Escribano Subias, P. Gomez Sanchez, M. A. Ruiz Cano, M. J. Severe biventricular hypertrophy and congestive heart failure resulting in a combined heart and hepatic transplantation. *European Journal of Heart Failure, Supplement.* 2012;1):S152. | CTS |
| Sahin EP, Y. Durmus-Tekçe, H. Cakar, A. A Val30Met sporadic familial amyloid polyneuropathy case with atypical presentation: Upper limb onset of symptoms. *Orphanet Journal of Rare Diseases. Conference: 1st European Meeting for ATTR Amyloidosis for Doctors and Patients. Paris France.* 2017;12. | CTS |
| Salutto VLA, V. C. Barroso, F. Mazia, C. G. Phenotypes of early-vs. Late-onset TTR MET30 familial amyloid polyneuropathy. *J Peripher Nerv Syst.* 2013;2):S101. | CTS |
| Salvalaggio AC, M. Tiengo, C. Angelini, A. Gasparotti, R. Briani, C. Multimodal evaluation of carpal tunnel syndrome in a pre-symptomatic TTR mutation carrier. *J Peripher Nerv Syst.* 2021;26(3):427. | CTS |
| Satterthwaite A. Carpal tunnel, a precursor to cardiomyopathy. *J Gen Intern Med.* 2019;34(2 Supplement):S503. | CTS |
| Sekijima YC, R. I. Hammarstrom, P. Nilsson, K. P. Yoshinaga, T. Nagamatsu, K. Yazaki, M. Kametani, F. Ikeda, S. Pathological, biochemical, and biophysical characteristics of the transthyretin variant Y114H (p.Y134H) explain its very mild clinical phenotype. *J Peripher Nerv Syst.* 2015;20:372-379. | CTRS |
| Slama MP, E. Cauquil, C. Algalarrondo, V. Eliahou, L. Labeyrie, C. Adams, D. Rouzet, F. Early detection of hTTR cardiac amyloidosis with bone DPD scintigraphy in a patient with a rare transthyretin mutation. *Eur J Heart Fail.* 2019;21(Supplement 1):343-344. | CTS |
| Sreedhar SS, R. Russell, S. Ashraf, T. Jafri, S. M. Gifting your entire liver to another: A unique case of domino liver transplantation. *Am J Gastroenterol.* 2020;115(SUPPL):S1283. | CTRS |
| Subramaniam PDLH, D. Corlett, R. J. Focal tenosynovial amyloid deposition as a rare cause of median nerve compression at the wrist. *Australian and New Zealand Journal of Surgery.* 1997;67(2-3):138-139. | CTS |
| Takei YH, T. Gono, T. Tokuda, T. Saitoh, S. Hoshii, Y. Ikeda, S. Senile systemic amyloidosis presenting as bilateral carpal tunnel syndrome. *Amyloid.* 2002;9:252-255. | CTS |
| Takei YH, T. Yazaki, M. Tokuda, T. Urasawa, N. Kanai, S. Ikeda, S. Transthyretin Tyr69-to-Ile mutation (double-nucleotide substitution in codon 69) in a Japanese familial amyloidosis patient with cardiomyopathy and carpal tunnel syndrome. *Amyloid.* 2003;10:25-28. | CTS |
| Telukuntla KSK, A. Kanj, M. Jaber, W. Hanna, M. Jacob, M. S. Manifestations of A Rare Italian Variant of Transthyretin Amyloidosis. *J Card Fail.* 2020;26(10 Supplement):S60-S61. | CTS |
| Tojo KT-S, A. Sekijima, Y. Morita, H. Sumita, N. Ikeda, S. I. Upper limb neuropathy such as carpal tunnel syndrome as an initial manifestation of ATTR Val30Met familial amyloid polyneuropathy. *Amyloid.* 2010;17(1):32-35. | CTS |
| Tong MSP, J. R. Khalil, R. Susco, B. Shah, M. Biederman, R. Cardiac amyloidosis masquerading as severe low-flow, low-gradient, low-ejection fraction aortic stenosis: "All that glitters is not gold". *J Am Coll Cardiol.* 2017;69(11 Supplement 1):2156. | CTS |
| Triguero AG-C, J. Lopez-Marne, S. Llop, A. Pane, M. Yun, S. Hand surgeons and amyloidosis specialists warning: transthyretin-associated amyloidosis with bifid median nerve as a cause of bilateral carpal tunnel syndrome. A case report and literature review. *Eur.* 2021;29:29. | CTS |
| Tsukada TT, M. Miyazaki, Y. Nishiura, Y. Yamashita, T. Kishikawa, M. A case of unilateral shoulder joint hydrarthrosis with wild-type amyloidogenic transthyretin amyloidosis. *Mod Rheumatol Case Rep.* 2020;4:312-317. | CTS |
| Tsunemitsu AS, S. Carpal tunnel syndrome due to amyloidosis. *Postgrad Med J.* 2021;97:61. | CTS |
| Uemichi TG, M. A. Benson, M. D. Amyloid polyneuropathy in two German-American families: a new transthyretin variant (Val 107). *J Med Genet.* 1994;31:416-417. | CTS |
| Uemichi TG, M. A. Benson, M. D. A new transthyretin variant (Ser 24) associated with familial amyloid polyneuropathy. *J Med Genet.* 1995;32:279-281 | CTS |
|  |  |
| Wolf SK, H. Hattr amyloidosis in a celiac disease patient. *Muscle and Nerve.* 2018;58(Supplement 2):S76. | CTS |
| Yadav MG, H. Nicu, M. Kamalakkannan, G. Vittorio, T. Krim, N. Bella, J. Severe Aortic Stenosis with Concurrent Transthyretin Amyloidosis. *J Am Coll Cardiol.* 2021;77(18 Supplement 1):2410. | CTS |
| Yamamoto HH, T. Kawamura, S. Hiroe, M. Yamashita, T. Ando, Y. Yokochi, T. Hereditary cardiac amyloidosis associated with Pro24Ser transthyretin mutation: a case report. *J Med Case Reports.* 2018;12:370. | CTS |
| Zolyomi ZB, M. D. Halasz, K. Uemichi, T. Fekete, G. Transthyretin mutation (serine 84) associated with familial amyloid polyneuropathy in a Hungarian family. *Amyloid.* 1998;5:30-34. | CTS |
| Fermin DRC, S. D. Twydell, P. T. Dickinson, M. G. Early Recurrence of Myocardial Transthyretin Amyloid Deposition Three Years Post Heart Transplantation for Hereditary V40I Amyloidosis. *J Card Fail.* 2019;25(8 Supplement):S157. | SS |
| Harats NW, R. Benson, M. D. Spinal claudication in systemic amyloidosis. *J Rheumatol.* 1989;16:1003-1006. | SS |
| Higashi HI, K. Ishibashi-Ueda, H. Yamaguchi, O. Hereditary Transthyretin Cardiac Amyloidosis in Refractory Spinal Canal Stenosis: Genetic and Pathologic Analysis. *Can J Cardiol.* 2021;37(8):1289-1291. | SS |
| Honig SM, R. Spinal cord claudication from amyloid deposition. *J Rheumatol.* 1992;19:1988-1990. | SS |
| Merve AP, R. A Case of fatal non-familial transthyretin amyloid deposition of the cervical spine. *Neuropathology and Applied Neurobiology.* 2017;43(Supplement 1):52. | SS |
| Polydefkis MN, S. Doherty, L. Ebenezer, G. J. Diagnostic challenges in amyloid neuropathies. *J Peripher Nerv Syst.* 2017;22(3):360-361. | SS |
| Reznik EVN, T. L. Borisovskaya, S. V. Brylev, L. V. Zhelnin, A. V. Seksyaev, N. E. A Clinical Case of the Hereditary Transthyretin Amyloidosis. *Russian Archives of Internal Medicine.* 2021;11(3):229-240. | SS |
| Sueyoshi TU, M. Sei, A. Misumi, Y. Oshima, T. Yamashita, T. Obayashi, K. Shinriki, S. Jono, H. Shono, M. Ando, Y. Mizuta, H. Spinal multifocal amyloidosis derived from wild-type transthyretin. *Amyloid.* 2011;18:165-168. | SS |
| Tan BEXT, S. Parikh, V. Concurrent Transthyretin Cardiac Amyloidosis and Light Chain Amyloidosis of the Foregut. *J Card Fail.* 2020;26(10 Supplement):S100. | SS |
| Kurkul AL, S. A puzzle finally solved. *J Am Geriatr Soc.* 2021;69(SUPPL 1):S38. | CTS + SS |
| Qiao JK, M. A Serendipitous Case of Classic ATTR Cardiac Amyloidosis (out of Sight out of Mind). *J Am Coll Cardiol.* 2021;77(18 Supplement 1):2151. | CTS + SS |
| Safren LM, A. Kadakkal, A. Sheikh, F. A Rare Case of Infiltrative Cardiomyopathy: Diagnostic Dilemma. *J Card Fail.* 2020;26(10 Supplement):S109-S110. | CTS + SS |
| Anan IB, J. Lundgren, H. E. Wixner, J. Westermark, P. A case report of osteoarthritis associated with hereditary transthyretin amyloidosis ATTRV30M. *Amyloid.* 2019;26:29-30. | OA |
| Boyadzhieva VS, N. Krasimirova-Kurteva, E. A case report of a patient with severe musculoskeletal manifestations of familial amyloid polyneuropathy and osteoarthritis. *Osteoporos Int.* 2019;30(SUPPL 2):S596. | OA |
| Reyes CMR, A. Kloss, R. Girardi, M. Lazova, R. Scleroderma-like illness as a presenting feature of multiple myeloma and amyloidosis. *J.* 2008;14:161-165. | OA |
| Takashio SN, M. Tsuruta, Y. Tsujita, K. Wild-type transthyretin amyloid cardiomyopathy complicated by spinal canal stenosis, carpal tunnel syndrome, and rotator cuff tears: a case report. *Eur Heart J Case Rep.* 2020;4:1-6. | CTS + SS + RCT |
| Uotani KK, A. Nagao, M. Mizutani, T. Hayashi, H. Trigger finger as an initial manifestation of familial amyloid polyneuropathy in a patient with Ile107Val TTR. *Intern Med.* 2007;46:501-504. | CTS + TF |
| Barge-Caballero GG-F, P. Barge-Caballero, E. Crespo-Leiro, M. G. Popeye's sign, heart disease, and amyloidosis. *Lancet.* 2019;393:e32. | NA* |
| Chaulagain CPC, R. L. Stigmata of amyloidosis; external manifestations of internal disease. *Br J Haematol.* 2019;186(1):10. | NA* |
| Damy TP-B, V. Dogan, A. Characterization of untyped cardiac amyloidosis by mass spectrometry in a patient with Gly6Ser transthyretin polymorphism in fatal cardiogenic shock. *Archives of Cardiovascular Diseases.* 2014;107(12):706-708. | NA* |
| Ikebe ST, S. Nishi, M. Morioka, M. Tsujita, K. Transthyretin Amyloid Cardiomyopathy Diagnosed on Incidental Myocardial Uptake During Bone Scintigraphy. *Circ J.* 2020;84:679. | NA* |
| Varedi DK, T. Downs Kelly, E. Abraham, J. Cowley, J. Barrell, K. Revelo, M. P. Stehlik, J. Drakos, S. Marrouche, N. Wilson, B. Swanson, E. A. Fang, J. Nativi-Nicolau, J. Unmasking early wild-type transthyretin amyloidosis cardiomyopathy in a patient with refractory atrial fibrillation and unremarkable cardiac imaging. *Circulation: Heart Failure.* 2018;11(7) (no pagination). | NA* |
| Yoshinaga TY, M. Ohno, M. Kodama, S. Koyama, J. Sekijima, Y. Cardiac amyloidosis associated with amyloidogenic transthyretin V122I variant in an elderly Japanese woman. *Circ J.* 2017;81(6):893-894. | NA* |

CTS = carpel tunnel syndrome; CTRS carpel tunnel release surgery; NA = not available; OA = osteoarthritis; RCT = rotator cuff tear; SS = spinal stenosis; TF = trigger finger. *Note case reports were excluded prior to full text review therefore specific MSK manifestation was not extracted from the full text.
